# Bone marrow-derived mesenchymal stem cells improve diabetes-induced cognitive impairment by exosome transfer into damaged neurons and astrocytes

**DOI:** 10.1038/srep24805

**Published:** 2016-04-22

**Authors:** Masako Nakano, Kanna Nagaishi, Naoto Konari, Yuki Saito, Takako Chikenji, Yuka Mizue, Mineko Fujimiya

**Affiliations:** 1 Department of Anatomy, Sapporo Medical University, School of Medicine, Sapporo, Hokkaido 060-8556, Japan

## Abstract

The incidence of dementia is higher in diabetic patients, but no effective treatment has been developed. This study showed that rat bone marrow mesenchymal stem cells (BM-MSCs) can improve the cognitive impairments of STZ-diabetic mice by repairing damaged neurons and astrocytes. The Morris water maze test demonstrated that cognitive impairments induced by diabetes were significantly improved by intravenous injection of BM-MSCs. In the CA1 region of the hippocampus, degeneration of neurons and astrocytes, as well as synaptic loss, were prominent in diabetes, and BM-MSC treatment successfully normalized them. Since a limited number of donor BM-MSCs was observed in the brain parenchyma, we hypothesized that humoral factors, especially exosomes released from BM-MSCs, act on damaged neurons and astrocytes. To investigate the effectiveness of exosomes for treatment of diabetes-induced cognitive impairment, exosomes were purified from the culture media and injected intracerebroventricularly into diabetic mice. Recovery of cognitive impairment and histological abnormalities similar to that seen with BM-MSC injection was found following exosome treatment. Use of fluorescence-labeled exosomes demonstrated that injected exosomes were internalized into astrocytes and neurons; these subsequently reversed the dysfunction. The present results indicate that exosomes derived from BM-MSCs might be a promising therapeutic tool for diabetes-induced cognitive impairment.

Cognitive impairment associated with diabetes mellitus is a worldwide problem, since epidemiological studies have reported that the incidence of dementia in diabetic patients is two- to three-fold higher than in non-diabetic persons^1^,^2^. Various mechanisms have been considered to cause diabetes-associated dementia, including glucose metabolism abnormalities such as hyperglycemia and hypoglycemia, insulin action abnormalities such as insulin deficiency and insulin resistance, vascular abnormalities and oxidative stress in the central nervous system (CNS)^3^,^4^. However similar to other diabetic complications, correcting these abnormalities does not necessarily improve the cognitive impairment^5^. In animal studies, various impairments of the CNS associated with type 1 diabetes have been reported, including neuronal damage by oxidative stress, decreased hippocampal synaptic plasticity, changes in glutamate neurotransmission, and dysfunctional swollen astrocytes that cause abnormal neuronal activities^6^,^7^,^8^,^9^. The type 2 diabetic model exhibits mitochondrial dysfunction, decreased synaptic integrity, swollen astroglial end-foot processes, and reduced insulin signaling in the hippocampus^10^,^11^,^12^. Therefore, the most important problems causing cognitive impairments in diabetes can be considered to be damaged neurons and astrocytes in the hippocampus.

The functional mechanisms of mesenchymal stem cells (MSCs) in the repair of damaged tissues, suppression of inflammatory responses, and modulation of the immune system have become the focus for their use in treating various diseases^13^,^14^. In our previous study, we resolved diabetes-induced hepatocyte damage by intravenous administration of bone marrow-derived MSCs (BM-MSCs) to streptozotocin (STZ)- and high fat diet- induced diabetic mice^15^. The results showed that BM-MSC-conditioned medium demonstrated similar effects to BM-MSCs themselves, suggesting that soluble factors derived from BM-MSCs might be responsible. Because BM-MSC migration and differentiation at target sites are very low and transient, it can be assumed that secretory factors, including exosomes derived from BM-MSCs, are the main therapeutic factors affecting damaged tissues.

Exosomes are cell-secreted membrane vesicles, sized 40 to 100 nm, that contain functional messenger RNAs (mRNAs) and micro RNAs (miRNAs), as well as proteins^16^,^17^. Exosomes are released from various cells and exert physiological actions through cell-cell communication^16^. In the CNS, neuron-derived exosomes play roles in regulating astroglial glutamate transporter-1 (GLT1) expression^18^, and they act on synaptic plasticity^19^. Astrocyte-derived exosomes, on the other hand, play roles in neuroprotection^20^ and promote neurite outgrowth and neuronal survival^21^. MSC-derived exosomes have been identified as therapeutic agents in MSC secretomes to reduce tissue injury and enhance tissue repair in cases of cardiovascular disease, lung injury, acute kidney injury, and neurovascular impairment after stroke^22^,^23^,^24^,^25^. In a stroke model, BM-MSC-derived exosomes have been shown to improve functional recovery and increase neurite remodeling, neurogenesis, and angiogenesis^25^. Therefore, it is conceivable that BM-MSC-derived exosomes can recover neuronal and astroglial damage induced by various causes.

Since, to our knowledge, no previously reported studies have shown the effects of BM-MSCs on diabetes-induced cognitive impairment, we investigated whether intravenously injected BM-MSCs can reverse neuronal and astroglial damage caused by diabetes and ameliorate cognitive impairment in diabetic mice. Furthermore, to examine the underlying mechanism of BM-MSC treatment, exosomes were isolated from the media of BM-MSC cultures and injected intracerebroventricularly, and the effects on neuronal and astroglial damage caused by diabetes were investigated. The results of the present study may suggest a novel therapeutic intervention for diabetes-induced cognitive impairment.

## Results

### BM-MSC administration ameliorated learning and memory impairment in diabetic mice

**Figure 1:**
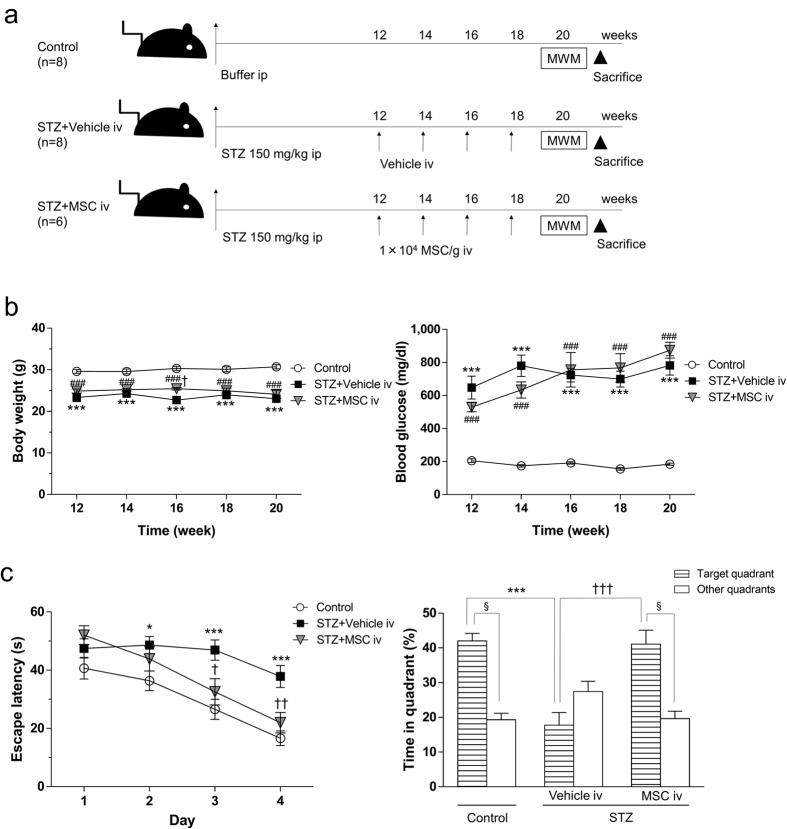
Intravenous injection of BM-MSCs into STZ-induced diabetic mice. (**a**) Experimental protocol. At 12 weeks after STZ injection, mice are injected iv with 1 × 10^4^ MSCs/g body weight four times every 2 weeks or with PBS for vehicle injection. Two weeks after the last injection, the Morris water maze (MWM) test is carried out. (**b**) Changes in body weight and serum blood glucose levels after MSC injection. ***P < 0.001, STZ + vehicle vs. control; ^###^*P* < 0.001, STZ + MSC vs. control; ^†^*P* < 0.05, STZ + vehicle vs. STZ + MSC, two-way ANOVA, Bonferroni post-test. Values are means ± s.e.m, n = 6–8. (**c**) MWM test. In the hidden training test, STZ + vehicle mice exhibit longer escape latency on days 2, 3 and 4 than control mice. The escape latency of STZ + MSC mice is shortened on days 3 and 4 compared to STZ + vehicle mice. *P < 0.05, ***P < 0.001, STZ + vehicle vs. control; ^†^*P* < 0.05, ^††^*P* < 0.01, STZ + vehicle vs. STZ + MSC, two-way ANOVA (*F*(2, 85) = 16.38 *P* < 0.0001). Values are means ± s.e.m, n = 6-8. In the probe test, the target quadrant occupancy of STZ + vehicle mice is significantly reduced compared with control mice (***P < 0.001, one-way ANOVA, Bonferroni post-test). The target quadrant occupancy of STZ + MSC mice is significantly elevated compared to STZ + vehicle mice. (^†††^*P* < 0.001, one-way ANOVA, Bonferroni post-test). Control and STZ + MSC mice spend significantly more time in the target quadrant than any of the other quadrants (^§^*P* < 0.0001, unpaired two-tailed *t*-test). For STZ + vehicle mice, the time spent in the target quadrant is similar to that spent in the other quadrants (*P* = 0.1406, unpaired two-tailed *t*-test).

No learning and memory impairments were observed at 8 weeks after STZ injection of mice (Suppl. Fig. 1a) but were observed at 12 weeks after STZ injection (Suppl. Fig. 1b). Therefore, in the present study, intravenous administration of 1 × 10^4^ BM-MSCs/g body weight was performed from 12 weeks after STZ injections, 4 times at 2-week intervals. The mice were then subjected to Morris water maze (MWM) tests and morphological analysis (Fig. 1a). During these experimental days, from 12 to 20 weeks after STZ injections, almost no changes were found in body weight and blood glucose levels between STZ + vehicle and STZ + MSC groups (Fig. 1b). In the STZ + vehicle mice, blood insulin levels were not detectable (Suppl. Fig. 2a) and blood glycated albumin levels were significantly higher compared to control mice (Suppl. Fig. 2b). In the hidden platform training session of the MWM tests, STZ + vehicle mice showed longer escape latency than control mice on days 2, 3 and 4. In STZ mice with MSC injection, the time of escape latency was significantly shortened compared to the STZ + vehicle group on days 3 and 4 (Fig. 1c). No difference was found in the swimming speed among the three experimental groups (controls; 0.1621 ± 0.0059 ms^−1^, STZ + vehicle; 0.1434 ± 0.0058 ms^−1^, STZ + MSC; 0.1364 ± 0.0091 ms^−1^). No difference was found in muscle endurance between control and STZ mice by the Hanging Wire test (Suppl. Fig. 3). In addition, no difference was found in the performance of the visible platform training session among the three groups, suggesting that sensorimotor function was not altered in STZ mice. During the probe trial, the time spent in the target quadrant was reduced in STZ + vehicle mice. In contrast, STZ + MSC mice spent longer times in the target quadrant, which implies retrieval of spatial memory (Fig. 1c).

We injected BM-MSCs into normal mice, 4 times at 2-week intervals, through the tail vein, and the MWM test was conducted. In the hidden training and probe test, there were no differences in escape latency and time spent in target quadrant time between the Control + vehicle and Control + MSC groups (Suppl. Fig. 4a,b). Further we injected BM-MSCs to diabetic mice at 12 weeks after STZ injection for 1 time, and the mice were subjected to MWM test at 1 week after BM-MSCs injection (Suppl. Fig. 5a). In the hidden training and probe test, there was significant difference in escape latency and time spent in target quadrant time between control and STZ + vehicle group, but no difference was detected between STZ + vehicle and STZ + MSC group (Suppl. Fig. 5b).

### BM-MSC-derived exosome administration ameliorated learning and memory impairment in diabetic miceh

Because a limited number of BM-MSCs was observed in the brain parenchyma after injection (Suppl. Fig. 6), we hypothesized that humoral factors, especially exosomes released from BM-MSCs, act on damaged neurons and astrocytes. To investigate the effectiveness of exosome treatment of diabetes-induced cognitive impairment, exosomes were purified from the BM-MSC culture media and injected intacerebroventricularly (icv) into STZ mice.

**Figure 2:**
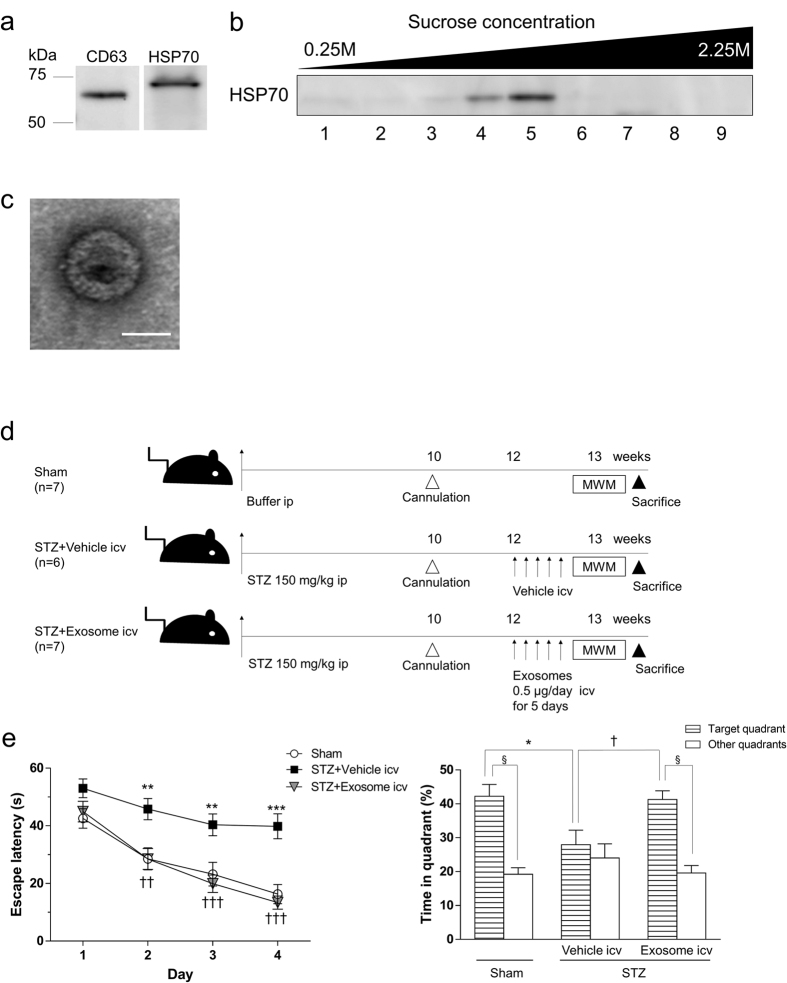
Isolation of BM-MSC-derived exosomes and intracerebroventricular injection of BM-MSC-derived exosomes into STZ-induced diabetic mice. (**a**) Western blot analysis for pellets obtained after 100,000 × g centrifugation. Positive reactions for CD63 and HSP70 are detected. (**b**) Further purification by a sucrose step gradient. HSP70 is detected at ranges between 1.11–1.15 g/mL (fractions 4–5). (**c**) Electron microscopic observation of an exosome. Bar, 50 nm. (**d**) Experimental protocols. At 12 weeks after STZ injection, mice are injected icv with 0.5 μg of exosomes in 2 μL aCSF once a day for 5 successive days, or with 2 μL aCSF for vehicle injection. Two days after the last injection, MWM tests are carried out. (**e**) MWM test. In the hidden training test, STZ + vehicle mice exhibit longer escape latency on days 2, 3 and 4 than sham-operated mice. The escape latency of STZ + exosome mice is shortened on days 2, 3 and 4 compared to STZ + vehicle mice. **P < 0.01, ***P < 0.001 STZ + vehicle vs. sham; ^††^*P* < 0.01, ^†††^*P* < 0.001, STZ + vehicle vs. STZ + exosome; two-way ANOVA (*F*(2,77) = 23.65, *P* < 0.0001). Values are means ± s.e.m, n = 6–7. In the probe test, the target quadrant occupancy of STZ + vehicle mice is significantly reduced (*P < 0.05, one-way ANOVA, Bonferroni post-test). The target quadrant occupancy of STZ + exosome mice is significantly higher than of STZ + vehicle mice. (^†^*P* < 0.05, one-way ANOVA, Bonferroni post-test). Sham and STZ + exosome mice spend significantly more time in the target quadrant than any of the other quadrants (^§^*P* < 0.0001, unpaired two-tailed *t*-test). For STZ + vehicle mice, the time spent in the target quadrant is similar to that spent in the other quadrants (*P* = 0.1406, unpaired two-tailed *t*-test).

To examine whether exosomes were successfully purified from culture media of BM-MSCs, Western Blot analysis for common protein markers of exosomes and electron microscopic analysis were performed. The pellets obtained after 100,000 × g centrifugation were positive for both CD 63 and HSP 70 (Fig. 2a), and after further purification by a sucrose step gradient followed by re-centrifugation, positive reactions for HSP 70 were detected at ranges between 1.11-1.15 g/mL, which is consistent with a previous report^26^ (Fig. 2b). These positive fractions (fractions 4 and 5) were collected as the final products. The cropped images of western blots displayed in the figure and the full-length blots were shown in Suppl. Fig. 7. Electron microscopic observations of the final products showed vesicles with diameters of 40–100 nm, which are characteristic for exosomes (Fig. 2c).

In the present study, exosomes purified from culture media of BM-MSCs were injected icv for 5 successive days at 12 weeks after STZ injection, and the mice were subjected to MWM tests (Fig. 2d). During these experimental days between 12 and 13 weeks after STZ injections, no changes were found in body weight and blood glucose levels (data not shown). In the hidden platform training session of the MWM tests, STZ + vehicle mice showed longer escape latency on days 2, 3 and 4 compared to sham-operated mice. In STZ mice with exosome injection, the time of escape latency was significantly shorter than for the STZ + vehicle group on days 2, 3 and 4 (Fig. 2e). No difference was found in the swimming speed among the three experimental groups (sham; 0.1657 ± 0.0062 ms^−1^, STZ + vehicle; 0.1588 ± 0.0073 ms^−1^, STZ + exosomes; 0.1664 ± 0.0053 ms^−1^). No difference was found in the performance of the visible platform training session among the three experimental groups. During the probe trial, the time spent in the target quadrant was reduced in STZ + vehicle mice. In contrast, STZ + exosome mice spent longer times in the target quadrant (Fig. 2e).

### BM-MSCs and BM-MSC-derived exosome administration does not increase the numbers of neurons but inhibits oxidative stress and increases synaptic density

**Figure 3:**
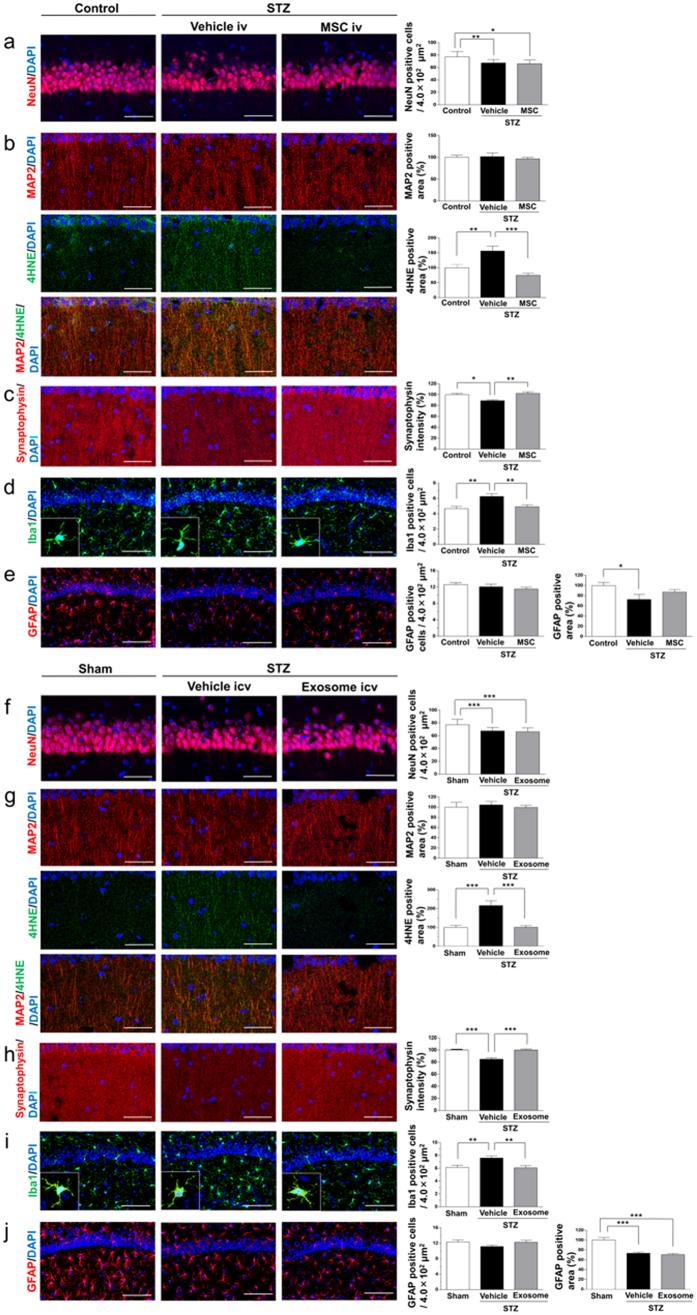
Morphological analysis in the CA1 region. (**a,f**) The number of NeuN-positive cells in STZ + vehicle mice is significantly decreased compared to control mice, and the number is not recovered in STZ + MSC and STZ + exosome mice. Bar, 50 μm. (**b,g**) The staining intensity of 4HNE(4-hydroxynonenal), an oxidative stress marker, is significantly increased in the dendrites (labeled with MAP2) of CA1 neurons in STZ + vehicle mice, and the increase is recovered to normal levels in STZ + MSC and STZ + exosome mice. There is no difference in the MAP2 area among the three groups. Bar, 50 μm. (**c,h**) The density of synaptophysin is significantly decreased in STZ + vehicle mice, and this decrease is recovered in STZ + MSC and STZ + exosome mice. Bar, 50 μm. (**d,i**) The number of Iba1-positive microglia is significantly increased in the STZ + vehicle group, and this increase recovers to normal levels in STZ + MSC and STZ + exosome mice. Bar, 100 μm. (**e,j**) The number of GFAP-positive astrocytes is not different among the three groups, but the area of the GFAP-positive reaction is decreased in STZ + vehicle mice. This decrease is not recovered in STZ + MSC mice nor in STZ + exosome mice. Bar, 100 μm. (**a–j**) *P < 0.05, **P < 0.01, ***P < 0.001, one-way ANOVA, Bonferroni post-test. Values are means ± s.e.m, n = 3–4.

In the next studies, the mechanism of how BM-MSCs and BM-MSC-derived exosomes ameliorated diabetes-induced cognitive impairment was examined. The numbers of pyramidal neurons in the CA1 region were compared (Fig. 3a,f). The density of NeuN-positive cells was significantly lower in the STZ + vehicle group than in control or sham mice, and the density was not recovered in the STZ + MSC and STZ + exosomes group (Fig. 3a,f). Therefore, no increase in neuronal cell number was detected in mice with MSC and exosome treatments.

In contrast, the staining positive area of 4HNE (4-hydroxynonenal), an oxidative stress marker, was significantly increased in the dendrites (labeled with MAP2) of CA1 neurons in the STZ + vehicle group, and the increase was recovered to normal levels in the STZ + MSC and STZ + exosomes group (Fig. 3b,g). In addition, the intensity of synaptophysin, which is expressed at presynaptic membranes and vesicles, and is correlated with synapse number and synaptic plasticity^27^, was significantly decreased in the STZ + vehicle group, but the decrease was recovered by MSC and exosome treatments (Fig. 3c,h). These results suggest that MSC and exosome treatments ameliorated the decrease in synaptic formation in the CA1 region caused by oxidative stress in diabetic mice.

### Effects of BM-MSC and BM-MSC-derived exosome treatments on microglia and astrocytes

Effects of BM-MSC and exosome treatments on microglia and astrocytes were examined by immunohistochemistry for microglia marker Iba1 and astrocyte marker GFAP in CA1. The results showed that the number of Iba1-positive microglia was significantly increased in the STZ + vehicle group, and this increase was recovered to normal levels in the STZ + MSC and STZ + exosomes group (Fig. 3d,i). Although it is known that microglia change from the resting state with a ramified shape into the activated state with a round shape by inflammatory stimulation, the present results showed no changes in morphology among the three groups, as the ramified shape was kept (Fig. 3d,i, enclosed by rectangle). This was confirmed by the quantitative analysis of the degree of ramification (Suppl. Fig. 8).

The number of GFAP-positive astrocytes in the CA1 region was not changed among the three groups, but the area of the GFAP-positive reaction was decreased in the STZ + vehicle group, and this decrease was not recovered by MSC or exosome treatment (Fig. 3e,j). To observe more details of the changes in neurons, as well as astrocytes, electron microscopic observations were performed in the three experimental groups.

### BM-MSC and BM-MSC-derived exosome treatments improve ultrastructural abnormalities induced by diabetes

**Figure 4:**
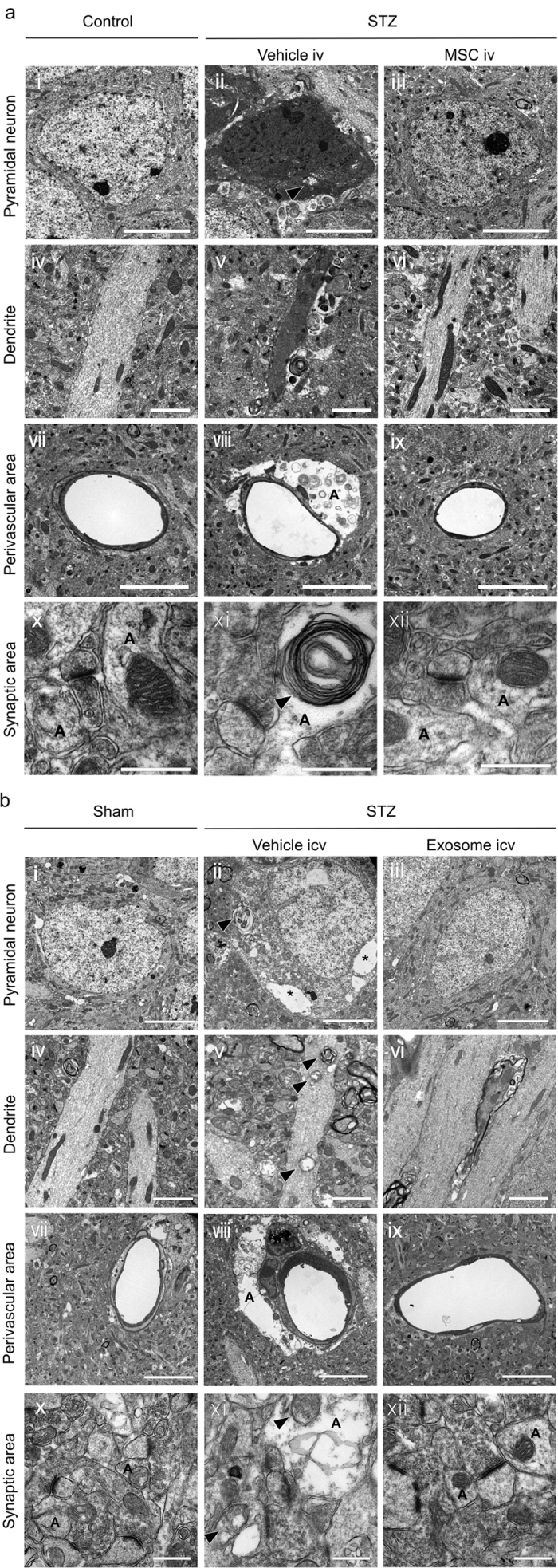
Electron microscopic study. (**a** i–iii) In the pyramidal layer, dark neurons with swollen mitochondria (arrowhead) are prominent in STZ + vehicle mice. These abnormalities have completely disappeared in STZ + MSC mice. Bar, 5 μm. (**b** i–iii) In the cytoplasm of pyramidal neurons, swollen mitochondria and vacuolization (*) are prominent in STZ + vehicle mice. These abnormalities have completely disappeared in STZ + exosome mice. Bar, 5 μm. (**a** iv–vi) While dark and shrunk dendrites are seen in STZ + vehicle mice, these abnormalities have completely disappeared in STZ + MSC mice. Bar, 2 μm. (**b** iv–vi) In dendrites, damaged mitochondria (arrowhead) and fragmentation of microtubules are seen in STZ + vehicle mice. These abnormalities have completely disappeared in STZ + exosome mice. Bar, 2 μm. (**a** vii–ix, **b** vii–ix) In the perivascular area of the CA1 region, astrocytic end-foot swelling (A) are prominent in STZ + vehicle mice. In addition, basement membrane of the blood vessels become irregular (**a** viii**, b** viii), and pericyte abnormality is found (**b** viii) in STZ + vehicle mice. These abnormalities have disappeared in STZ + MSC and STZ + exosome mice. Bar, 5 μm. (**a** x–xii, **b** x–xii) In the synaptic area, astrocytic end-foot swelling and abnormal mitochondria (arrowhead) in astrocytes (A) are prominent in STZ + vehicle mice. In addition, hypertrophy of the asymmetric synapses were observed in STZ + vehicle mice. However these abnormalities have completely recovered in STZ + MSC and STZ + exosome mice. Bar, 0.5 μm.

On electron microscopic study, massive abnormalities were found in the cytoplasm of pyramidal cells, dendrites and astrocytes in the STZ + vehicle mice. In the cytoplasm of pyramidal neurons in the STZ + vehicle mice, swollen mitochondria and vacuolization were prominent (Fig. 4aii, bii). In dendrites, damaged mitochondria and fragmentation of microtubules were seen in STZ + vehicle mice (Fig. 4bv). In late-stage STZ mice (20 weeks after STZ injection), damaged neurons with high electron density (dark neuron) were observed (Fig. 4aii, v); they were not seen in early-stage STZ mice (13 weeks after STZ injection) (Fig. 4bii, v). In the perivascular area of the CA1 region in STZ + vehicle mice, astrocytic end-foot process swelling was prominent (Fig. 4aviii, bviii). The basement membrane of the blood vessels became irregular (Fig. 4aviii, bviii), and pericyte abnormality was found (Fig. 4bviii). Astrocytic end-foot process swelling around axo-dendritic synapses, and hypertrophy of the asymmetric synapses were observed in STZ + vehicle mice (Fig. 4axi, bxi). However no change in the ultrastructure of microglia was found among the three groups (Suppl. Fig. 9). Surprisingly, the ultrastructural abnormalities in neurons, astrocytes, and blood vessels were completely recovered in mice treated with MSCs and exosomes (Fig. 4aiii, vi, ix, xii, biii, vi, ix, xii).

### Exosomes are internalized into neurons, astrocytes, and microglia

**Figure 5:**
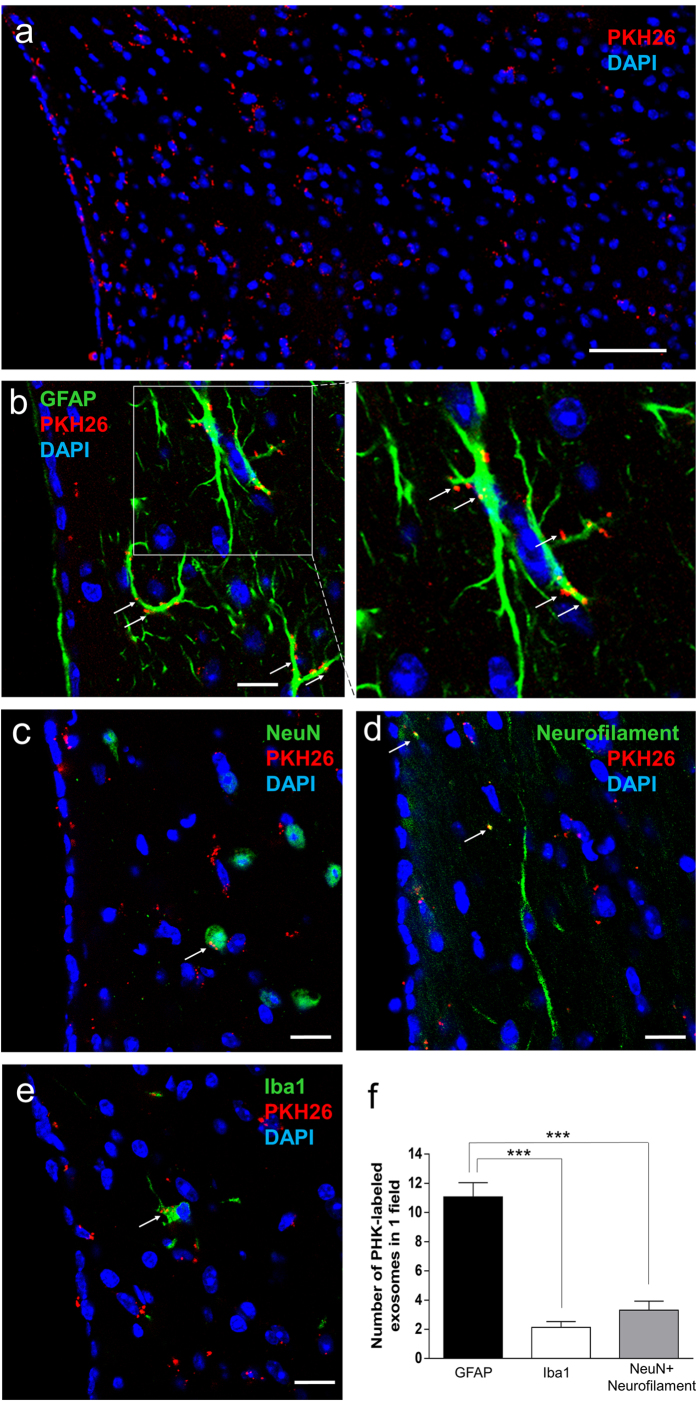
Distribution of icv injected exosomes in the brain parenchyma. (**a**) A number of PKH-labeled exosomes is observed in the brain parenchyma at the fimbria hippocampi. Bar, 50 μm. (**b**) A remarkable number of labeled exosomes is detected at the plasma membrane, as well as in the cytoplasm, of GFAP-positive astrocytes (arrows). Bar, 10 μm. (**c–e**) A few labeled exosomes are found in the cytoplasm of NeuN- and neurofilament-positive neurons and Iba1-positive microglia (arrows). Bar, 10 μm. (**f**) The number of PKH-labeled exosomes within GFAP-positive astrocytes is significantly larger than those within NeuN- and neurofilament-positive neurons or Iba1-positive microglia. ***P < 0.001, one-way ANOVA, Bonferroni post-test. Values are means ± s.e.m, n = 4.

To visualize the distribution of exosomes in the brain parenchyma after intracerebroventricular administration, exosomes were labeled with PKH26. Immunohistochemistry of the mouse brain revealed that a number of PKH-labeled exosomes was found in the brain parenchyma at the fimbria hippocampi (Fig. 5a), with a relatively small number at other periventricular areas (data not shown). A number of labeled exosomes was found at the plasma membrane, as well as in the cytoplasm, of GFAP-positive astrocytes (Fig. 5b). A few labeled exosomes were found in the cytoplasm of NeuN-positive neuronal cell bodies (Fig. 5c), neurofilament-positive axons/dendrites (Fig. 5d) and Iba1-positive microglia (Fig. 5e). The number of PKH-labeled exosomes within GFAP-positive astrocytes was significantly larger than that within NeuN- and neurofilament-positive neurons or Iba1-positive microglia (Fig. 5f).

## Discussion

The present study showed that intravenously injected BM-MSCs ameliorated diabetes-induced cognitive impairments and successfully repaired the damaged neurons and astrocytes in diabetic mice. Because a very small number of donor BM-MSCs was detected in the brain parenchyma after injection, we hypothesize that exosomes derived from BM-MSCs are the main therapeutic factors affecting damaged tissues.

Since learning and memory impairments were detected in diabetic mice at 12 weeks after STZ injection, treatment with BM-MSCs or exosomes was started at this time point. Intravenous injections of BM-MSCs were given 4 times at 2-week intervals, according to our previous study^15^, while intracerebroventricular injections of exosomes were given for 5 successive days. A previous report has described that IR Dye 800-labeled exosomes disappeared from the brain in 24 h after intranasal administration^28^; therefore, they were repeatedly applied every 24 h for 5 days to maintain higher concentrations in the brain. We used rat BM-MSCs for treatment of STZ mice because BM- MSCs are well known as “off-the-self” cells because of lack of MHC-II antigen^29^. The purity of isolated exosomes from culture media of BM-MSCs was examined by Western blotting for the common exosome markers CD63 and HSP70^30^,^31^. Sucrose gradients were used at ranges of 1.11–1.15 g/mL for exosome isolation, which was distinguished from ranges of 1.05–1.12 g/mL for isolation of Golgi body-derived vesicles or ranges of 1.18–1.25 g/mL for isolation of endoplasmic reticulum-derived vesicles^32^. HSP70-positive fractions were obtained at ranges of 1.11–1.15 g/mL, suggesting that exosomes were successfully purified from BM-MSC culture media. Exosomes (0.5 μg) in 2 μL aCSF were injected each day, because in a previous study, 4 μg exosomes derived from N2a cells in 5 μL PBS were injected icv in rats to inhibit the disrupting effects of Aβ on synaptic plasticity^33^. Considering the difference in CSF volume between rats and mice, approximately 90 μL and 10 μL, respectively^34^,^35^, the 0.5 μg exosomes used in the present study seem to be appropriate for intracerebroventricular injection in mice.

Both BM-MSC and exosome injections showed similar effects on the MWM test. STZ diabetic mice took significantly longer escape latency to find the platform, indicating a decrease of spatial learning ability. In the probe test, STZ mice spent less time in the platform quadrant, which indicates memory dysfunction. It seems obvious that the impaired performance of STZ mice is due to cognitive impairment rather than sensorimotor deficits, because there was no difference between control/sham and STZ mice in the visible platform test and Hanging Wire test which can measure the strength of upper and lower body muscle^36^. Both BM-MSC and exosome treatments ameliorated learning and memory impairment in STZ mice, even though hyperglycemic conditions remained. In experiments using normal mice, BM-MSCs injection showed no effects on cognitive functions. Therefore, BM-MSCs treatment can revert cognitive impairment selectively in STZ mice, but not enhance the cognitive function in normal mice. Icv-injection of 0.5 μg exosomes per day for 5 successive days reverted cognitive impairment in STZ mice only at 1 week after the injection, however 1 time iv-injection of 2.5 × 10^5^ BM-MSCs per mouse had no effects. Such discrepancy seems to be a difference in volume of exosome, 2.5 × 10^5^ BM-MSCs are estimated to include 0.1 μg of exosomes, and also a difference in injection rout, iv and icv.

The results showed that, in STZ mice, the number of NeuN-positive pyramidal neurons in the CA1 region of the hippocampus was decreased, while oxidative damage was increased more in dendrites than in neuropil, detected by overlap staining with 4HNE and MAP2. These results were consistent with previous studies^37^,^38^. In STZ mice, a reduction of the synaptophysin intensity was also observed, which is correlated with the synaptic number^27^,^39^. BM-MSC and exosome treatments ameliorated oxidative stress and enhanced synaptic number, but did not affect the number of pyramidal neurons. The area of MAP2-positive dendrites was not changed in spite of a decrease in the number of NeuN-positive neurons. Such discrepancy seems to be due to a compensatory increase of dendrites in hyperglycemic conditions^40^. It is known that MSCs are multipotent progenitors and are considered to differentiate into neurons or enhance endogenous neurogenesis^41^,^42^. However, in the present results, neither BM-MSCs nor exosomes altered the number of NeuN-positive neurons in STZ-diabetic mice, suggesting that injected BM-MSC or exosomes may restore the function of the remaining neurons and promote synaptogenesis after diabetic tissue damage. These results were consistent with previous studies which showed that memory loss correlates with synaptic loss but not with neuronal loss^39^,^43^, and therefore recovery of the number of synapses by BM-MSCs and exosomes treatments may contribute to improvement of cognitive impairment.

The effects of treatments with BM-MSCs or exosomes on glial cells including microglia and astroglia were examined. The number of Iba1-positive microglia was significantly increased in STZ mice compared to controls, and this increase was recovered to normal levels in both BM-MSC- and exosome-treated groups, suggesting that these treatments may suppress the proliferation of microglia in the brain. Although microglia are known to change from a resting state with a ramified shape into an activated state with a round shape by inflammatory stimuli including hyperglycemia^44^,^45^, the present results in all experimental groups showed that they kept the ramified shape. In fact the expression of IL-1β, which is known to up-regulated in activated microglia, was not changed between treated and non-treated groups (Suppl. Fig. 10). Therefore damaged neurons in the CA1 region seem to stimulate the proliferation of microglia, but after treatment such phenomena ceased in response to the decrease in damaged neurons. On the other hand, the number of GFAP-positive astrocytes in the CA1 region was not changed among the three groups, but the area of the GFAP-positive reaction was decreased in STZ mice, and this decrease was not altered in the BM-MSC-treated and exosome-treated groups. It has been reported that type 1 diabetes induces hypertrophic changes in astrocytes with up- or down-regulation of GFAP expression^45^,^46^,^47^,^48^. In type 2 diabetes, up-regulation of GFAP with synaptic alteration in the hippocampus was shown by Duarte *et al*.^49^; on the other hand, down-regulation of GFAP in the hippocampus was shown by Amin *et al*.^48^. Therefore, for further analysis of changes in neurons or astrocytes, electron microscopic observation of the brain parenchyma was performed.

Electron microscopic study revealed damaged mitochondria and vacuolation and fragmentation of microtubules in perikarya and dendrites of pyramidal neurons in the CA1 regions of STZ mice. In late-stage STZ mice, damaged neurons with high electron density characteristic of dark neurons were seen, and these are known to be generated by increased free radicals^50^. Surprisingly, these morphological abnormalities were completely recovered by either BM-MSC or exosome treatment. Because mitochondrial damage is correlated with oxidative stress, these structural abnormalities are consistent with increases in 4HNE immunoreactivity in neurons, as shown in the present study. In previous studies, type 2 diabetic mice showed the decrease in the expression of NRF2, an antioxidant defense factor in mitochondria, which correlates with the synaptic damage in the hippocampus^10^. Our treatment successfully normalized the damaged neurons, possibly caused by oxidative stress, and such remarkable recovery of CA1 neurons by both BM-MSCs and exosome treatments may lead to improvement of cognitive impairment as assessed by the MWM test. Morphological abnormalities in astrocytes were prominent in STZ mice, including mitochondrial damage and swollen end-feet around vessels and synapses. These abnormalities were completely restored by both BM-MSCs and exosome treatments in brain. Although the decrease in the GFAP-positive area was not recovered by MSC and exosome therapies, electron microscopy showed that these therapies improved astrocyte abnormalities. It seems that GFAP did not reflect the swollen end-foot, because GFAP is entirely absent from the finely branching astrocyte processes^51^. Astrocytes are known to swell with inflammation, and swollen astrocytes possess less power to take up excess glutamate, which has neurotoxic effects at synapses^52^. The present results suggest that functional impairments in astrocytes caused by diabetes were recovered by BM-MSC and exosome treatments.

To assess the mechanism whereby icv-injected exosomes improved neuronal and astroglial damage and reversed cognitive impairment, exosomes were labeled with PKH, and the distribution of injected exosomes was examined. PKH-labeled exosomes were detected at the fimbria hippocampi, consisting mainly of regularly arranged astroglial processes and longitudinal axes of axons^53^. These axons terminate on target neurons in the dentate gyrus and the cornu ammonis^54^. The present results showed that the majority of PKH-labeled exosomes were taken up by GFAP-positive astrocytes, and a few PKH-labeled exosomes were taken up by NeuN- and neurofilament-positive neuronal cells and Iba1-positive microglia. A previous study showed that PKH-labeled exosomes derived from choroid plexus cell lines penetrated into brain parenchyma and were initially taken up by astrocytes and secondarily delivered to neurons^55^. Another study showed that GFP-labeled exosomes derived from BM-MSCs were taken up by astrocytes and neurons after intravenous injection of BM-MSCs in a stroke model^56^.

Astrocytes are more abundant than neurons and form wide networks with each other through gap junctions^57^, and their thin processes contact cerebrospinal fluid to form glia-limitans at the surface of brain parenchyma^58^. Therefore icv-injected PKH-labeled exosomes may be easily taken up by astrocytes. To maintain brain homeostasis, astrocytes play important roles, including supplying lactate, an energy source for neurons, controlling extracellular K^+^ levels, and removing excess glutamate, which has neurotoxic effects on synapses^51^. Astrocytes are essential for maintenance of synapses and synaptogenesis^59^; in fact, individual astrocytes in the hippocampus touch up to 100,000 synapses^60^. In the present study, we detected the internalization of PKH-labeled exosomes only in a small area of the brain, because PKH-positive reactions were difficult to detect in expanded area due to their low density. However since previous study reported that exosomes can diffuse into a large area once they have entered the brain^28^, we considered that icv-injected exosomes might undergo widespread diffusion into a large area of the brain including the hippocampus and be taken up by neurons and astrocytes.

In the presents study, BM-MSCs were administered intravenously in STZ diabetic mice, and exosomes isolated from cultured BM-MSCs were administered icv, and both treatments successfully ameliorated the cognitive impairment caused by diabetes. The exosomes were internalized by astrocytes, as well as neurons, which led to the recovery of astrocytes and neurons damaged by diabetes. Exosomes secreted by BM-MSCs administered intravenously may easily diffuse into brain parenchyma from blood vessels and into the CSF^56^, then be internalized into astrocytes and neurons. Although the specific miRNAs or proteins involved in repairing damaged cells were not identified in this study, the present results indicate that transfer of exosomes released from intravenously administered BM-MSCs into damaged astrocytes and neurons have effects to repair damaged neurons and astrocytes. Especially, functionally recovered astrocytes might boost neuronal functions, brain homeostasis, and synaptogenesis.

The present results may open a new horizon for the treatment of cognitive impairment caused by not only diabetes but also presumably other causes that damage neurons and astrocytes. Systemic administration of BM-MSCs or local injection of exosomes obtained from cultured BM-MSCs might be a powerful therapeutic agent for diabetes-induced cognitive impairments.

## Methods

### Animals

All the protocols were carried out in accordance with the approved guidelines. C57BL/6 J mice were purchased from Clea Japan Inc (Tokyo, Japan). Hyperglycemia was induced by a single intraperitoneal injection (ip) of STZ (150 mg/kg; Wako, Osaka, Japan) dissolved in citrate buffer (pH 4.5) at 13 weeks of age. Controls were administered an intraperitoneal (ip) injection of citrate buffer. STZ-diabetic mice with blood glucose levels >300 mg/dL were used. This study was approved by the animal experiment committee of Sapporo Medical University (Sapporo, Japan).

### Isolation of bone marrow MSC

Rat BM-MSCs were harvested from bone marrow of 8-week-old SD rats (Sankyo Labo Service Corporation Inc, Tokyo, Japan) as described previously^15^. In brief, after the sacrifice of male SD rats, the tibias and femurs were removed and cleaned of connective tissues. Marrow was flushed out after removal of the epiphysis and cultured with alpha-minimal essential medium (Gibco, Life Technology Japan, Tokyo, Japan) containing 15% fetal bovine serum (CCB, NICHIREI BIOSCIENCE, Tokyo, Japan) and 1% penicillin streptomycin (Pen Strep, Life Technologies, Carlsbad, CA, USA). The medium was changed twice weekly, and BM-MSCs harvested in 3-4 passages were used for further study. We followed the procedure of BM-MSCs isolation used in our previous study; they were positive for CD90 and negative for CD45, CD11b, and could differentiate into osteoblasts, adipocytes and chondrocyte-like cells^15^.

### Intravenous injection of BM-MSCs

At 12 weeks after STZ injection, mice were administered BM-MSCs (1 × 10^4^ BM-MSCs/g body weight per animal suspended in 200 μl of PBS) through the tail vein, 4 times at 2-week intervals (Fig. 1a). The vehicle injection consisted of 200 μl of PBS. We also injected BM-MSCs (1 × 10^4^ BM-MSCs/g body weight) to diabetic mice at 12 weeks after STZ injection for 1 time, and injected to normal mice 4 times with 2-week intervals from tail vein.

### Isolation of bone marrow MSC-derived exosomes

Rat BM-MSCs were harvested as previously described. To isolate exosomes, conventional culture medium was replaced by a medium containing exosome-depleted fetal bovine serum (EXO-FBS-250 A-1, System Biosciences, Mountain View, CA, USA), according to a previous report^56^. When MSCs (passage 4) reached 60–80% confluence, the cells were cultured for an additional 48 h. The culture media were then collected, and the following procedures were performed for exosome isolation at 4 °C. The collected media were first centrifuged at 1,000 × *g* for 10 min to remove large debris and dead cells. Then, the supernatants were centrifuged at 10,000 × *g* for 30 min to remove small debris. To obtain pellets that contained exosomes, the supernatants were further centrifuged at 100,000 × g for 3 h. After discarding the supernatants, the pellets were resuspended in 0.25 M sucrose in 20 mM HEPES, pH 7.4, and loaded onto a sucrose step gradient (2.25, 2.0, 1.75, 1.5, 1.25, 1.0, 0.75 and 0.5 M). The sucrose gradient was then centrifuged at 100,000 × *g* for 16 h, and 9 fractions corresponding to each sucrose step gradient were collected and diluted with 20 mM HEPES, pH 7.4, and recentrifuged at 100,000 × *g* for 1 h. Each resulting pellet was resuspended in PBS and stored at −80 °C for further use.

### Cannulation surgery and intracerebroventricular injection of exosomes

At 10 weeks after STZ injection, mice were implanted with stainless steel cannula (0.4 mm diameter) into the right ventricle of the brain. Coordinates for cannulation were 0.4 mm posterior from the bregma, 1 mm lateral from the midline, and 2.5 mm depth (from the brain surface). For injection of exosomes we used a Hamilton syringe with a needle of 0.17 mm diameter. At 12 weeks after STZ injection, mice were injected icv with 0.5 μg of exosome in 2 μL artificial cerebrospinal fluid (aCSF; 142 mM NaCl, 5 mM KCl, 2 mM CaCl_2_ ∙2 H_2_O, 2 mM MgCl_2_ ∙ 6 H_2_O, 1.25 mM NaH_2_PO_4,_, 10 mM D-glucose, 10 mM HEPES, pH 7.4) once a day for 5 successive days, or with 2 μL aCSF as the vehicle injection. To examine protein concentrations of exosomes, the bicinchoninic acid protein assay (Thermo Fisher Scientific, Lafayette, CO, USA) was used.

### Morris water maze tests

The task was given at 9:00–13:00. The apparatus consisted of a circular pool (1.2 m diameter and 30 cm high) filled to a depth of 18 cm with water (25 ± 1 °C). A variety of visual cues were provided in the testing area and kept constant throughout the task process. Data were obtained from a video camera (Logicool, Tokyo, Japan), which was connected to an automated tracking system (Any-maze, Stoelting, Wood Dale, IL, USA), fixed 1.7 m above the center of the pool. A transparent acrylic platform (10 cm diameter) was placed in the center of one quadrant of the pool. In visible platform training (day 0), the test was performed to get mice used to the pool and to assess swimming ability and vision. The platform was placed 1 cm below the surface of the water, and a small flag (15 cm in height) was attached for visualization of the platform area. In the hidden platform training (day 1–4), the tasks were carried out to evaluate the extent of learning. During the training, the platform with no flag remained in the same location and was submerged 1 cm below the surface of the water. Mice performed 4 trials a day with a 1-h interval between trials. Each trial had a time limit of 60 sec, and mice had to swim until they climbed onto the platform. After reaching the platform, mice were allowed to remain for 15 sec. If they failed to find the platform within 60 sec, the test was ended, and the mice were gently placed on the platform by the experimenter for 15 sec. At day 5, the platform was removed, and the probe trial was conducted to assess the extent of memory. Each mouse was released from the start position opposite to the former platform quadrant. They were allowed 60 sec to search for the platform, and the time spent in the quadrant where the platform was previously located was recorded.

### Immunofluorescence staining

One day after the MWM, the mice were sacrificed for morphological studies. The mice were perfused with 0.1 M PBS via the left ventricle to wash out blood, followed by a solution of 4% paraformaldehyde (PFA), 0.2% picric acid, and 0.5% glutaraldehyde in 0.1 M phosphate buffer. Brains were removed and immersed in a solution of 4% PFA and 0.2% picric acid in 0.1 M phosphate buffer for 24 h and then immersed gradually in 5%, 10%, and 15% sucrose solutions. Frozen sections (20 μm) corresponding to sagittal coordinates extending 1.4–2.4 mm from the bregma in the left hemisphere were made. The sections were incubated in primary antibodies against NeuN (rabbit polyclonal, 1:1000; Millipore, Darmstadt, Germany), GFAP (chicken polyclonal, 1:500; Millipore), Iba1 (rabbit polyclonal, 1:500; Wako, Osaka, Japan), MAP2 (chicken polyclonal, 1:500; Millipore), 4-hydroxynonenal (4HNE) (rabbit polyclonal, 1:100; Abcam, Cambridge, UK), and synaptophysin (rabbit polyclonal, 1:500; Sigma-Aldrich, St. Louis, MO, USA), 200kD phosphorylated and non-phosphorylated neurofilament heavy (rabbit polyclonal, 1:500, Abcam), and IL-1β (hamster monoclonal, 1:500, Biolegend, San Diego, CA, USA). For second antibodies, Cy3-labeled anti-rabbit IgG (Jackson ImmunoResearch, West Grove, PA, USA), Cy3-labeled anti-chicken IgG (Millipore), Cy-5 labeled anti-hamster IgG (Jackson ImmunoResearch), and FITC-labeled anti-rabbit IgG (Millipore) diluted 1:500 were used. Nuclei were stained with DAPI (Dojindo, Kumamoto, Japan) and observed under confocal laser scanning microscopy (Nikon A1, Tokyo, Japan).

Quantitative analyses for the immuno-positive cells, area, and intensity were performed in three selected sections 240 μm apart through the CA1 region in control, sham-operated, STZ + vehicle, STZ + MSC, and STZ + exosome mice. A total of 9 different fields (200 × 200 μm) per mouse (3 fields per section) were analyzed by a Nikon software program (NIS Elements AR). To avoid contamination by background staining, we detected structures with more than 0.5 μm^2^ as immune-positive.

### Electron microscope observations

After perfusion fixation, the right hemispheres were cut into 100-μm-thick sections by a vibratome (VT12005, Leica, Tokyo, Japan). The sections corresponding to sagittal coordinates extending between 1.4–2.4 mm from the bregma were fixed with 2.5% glutaraldehyde, then post-fixed with 2% osmium tetroxide, and embedded in epon (EPON812, TAAB Laboratories Equipment, Berks, UK). Ultra-thin sections (70 nm) were cut with an ultramicrotome (RMT, Tucson, AZ, USA), stained with uranyl acetate and lead citrate, and observed under electron microscopy (H7650, HITACHI, Tokyo, Japan).

### Statistical Analysis

Data are expressed as means ± s.e.m. Statistical analysis was carried out using unpaired *t*-test or one-way ANOVA followed by Bonferroni’s test for post hoc comparisons between groups. When two factors were assessed, the significance of differences was determined using two-way ANOVA followed by Bonferroni’s test for post hoc comparisons between groups. Statistical analysis was performed using GraphPad Prism 6.0 (GraphPad Software Inc., San Diego, CA, USA), and differences were considered significant at *P* < 0.05.

## Additional Information

**How to cite this article**: Nakano, M. *et al*. Bone marrow-derived mesenchymal stem cells improve diabetes-induced cognitive impairment by exosome transfer into damaged neurons and astrocytes. *Sci. Rep.* **6**, 24805; doi: 10.1038/srep24805 (2016).

## Supplementary Material

Supplementary Information

## References

[b1] Leibson C. L. . Risk of dementia among persons with diabetes mellitus: a population-based cohort study. Am J Epidemiol 145, 301–308 (1997). 9054233 10.1093/oxfordjournals.aje.a009106

[b2] Ott A. . Diabetes mellitus and the risk of dementia: The Rotterdam Study. Neurology 53, 1937–1942 (1999). 10599761 10.1212/wnl.53.9.1937

[b3] Wrighten S. A., Piroli G. G., Grillo C. A. & Reagan L. P. A look inside the diabetic brain: Contributors to diabetes-induced brain aging. Biochim Biophys Acta 1792, 444–453 (2009). 19022375 10.1016/j.bbadis.2008.10.013 PMC3991007

[b4] Kawamura T., Umemura T. & Hotta N. Cognitive impairment in diabetic patients: Can diabetic control prevent cognitive decline? J Diabetes Investig 3, 413–423 (2012). 10.1111/j.2040-1124.2012.00234.x PMC4019239 24843599

[b5] Sakata A. . Improvement of cognitive impairment in female type 2 diabetes mellitus mice by spironolactone. J Renin Angiotensin Aldosterone Syst 13, 84–90 (2012). 21729993 10.1177/1470320311412810

[b6] Revsin Y. . Neuronal and astroglial alterations in the hippocampus of a mouse model for type 1 diabetes. Brain Res 1038, 22–31 (2005). 15748869 10.1016/j.brainres.2004.12.032

[b7] Hernandez-Fonseca J. P. . Structural and ultrastructural analysis of cerebral cortex, cerebellum, and hypothalamus from diabetic rats. Exp Diabetes Res 2009, 329632 (2009). 10.1155/2009/329632 PMC2756466 19812703

[b8] Aragno M. . Up-regulation of advanced glycated products receptors in the brain of diabetic rats is prevented by antioxidant treatment. Endocrinology 146, 5561–5567 (2005). 16166220 10.1210/en.2005-0712

[b9] Son H. . Type 1 diabetes alters astrocytic properties related with neurotransmitter supply, causing abnormal neuronal activities. Brain Res 1602, 32–43 (2015). 25578257 10.1016/j.brainres.2014.12.055

[b10] Carvalho C., Santos M. S., Oliveira C. R. & Moreira P. I. Alzheimer’s disease and type 2 diabetes-related alterations in brain mitochondria, autophagy and synaptic markers. Biochim Biophys Acta 1852, 1665–1675 (2015). 25960150 10.1016/j.bbadis.2015.05.001

[b11] Min L. J. . Peroxisome proliferator-activated receptor-gamma activation with angiotensin II type 1 receptor blockade is pivotal for the prevention of blood-brain barrier impairment and cognitive decline in type 2 diabetic mice. Hypertension 59, 1079–1088 (2012). 22454480 10.1161/HYPERTENSIONAHA.112.192401

[b12] Agrawal R. . Deterioration of plasticity and metabolic homeostasis in the brain of the UCD-T2DM rat model of naturally occurring type-2 diabetes. Biochim Biophys Acta 1842, 1313–1323 (2014). 24840661 10.1016/j.bbadis.2014.05.007 PMC4372388

[b13] Prockop D. J. & Oh J. Y. Mesenchymal stem/stromal cells (MSCs): role as guardians of inflammation. Mol Ther 20, 14–20 (2012). 22008910 10.1038/mt.2011.211 PMC3255583

[b14] Ma S. . Immunobiology of mesenchymal stem cells. Cell Death Differ 21, 216–225 (2014). 24185619 10.1038/cdd.2013.158 PMC3890955

[b15] Nagaishi K., Ataka K., Echizen E., Arimura Y. & Fujimiya M. Mesenchymal stem cell therapy ameliorates diabetic hepatocyte damage in mice by inhibiting infiltration of bone marrow-derived cells. Hepatology 59, 1816–1829 (2014). 24375439 10.1002/hep.26975

[b16] Thery C. Exosomes: secreted vesicles and intercellular communications. F1000 Biol Rep 3, 15 (2011). 21876726 10.3410/B3-15 PMC3155154

[b17] Thery C., Zitvogel L. & Amigorena S. Exosomes: composition, biogenesis and function. Nat Rev Immunol 2, 569–579 (2002). 12154376 10.1038/nri855

[b18] Morel L. . Neuronal exosomal miRNA-dependent translational regulation of astroglial glutamate transporter GLT1. J Biol Chem 288, 7105–7116 (2013). 23364798 10.1074/jbc.M112.410944 PMC3591620

[b19] Korkut C. . Regulation of postsynaptic retrograde signaling by presynaptic exosome release. Neuron 77, 1039–1046 (2013). 23522040 10.1016/j.neuron.2013.01.013 PMC3626103

[b20] Taylor A. R., Robinson M. B., Gifondorwa D. J., Tytell M. & Milligan C. E. Regulation of heat shock protein 70 release in astrocytes: role of signaling kinases. Dev Neurobiol 67, 1815–1829 (2007). 17701989 10.1002/dneu.20559

[b21] Wang S. . Synapsin I is an oligomannose-carrying glycoprotein, acts as an oligomannose-binding lectin, and promotes neurite outgrowth and neuronal survival when released via glia-derived exosomes. J Neurosci 31, 7275–7290 (2011). 21593312 10.1523/JNEUROSCI.6476-10.2011 PMC6622588

[b22] Bian S. . Extracellular vesicles derived from human bone marrow mesenchymal stem cells promote angiogenesis in a rat myocardial infarction model. J Mol Med 92, 387–397 (2014). 24337504 10.1007/s00109-013-1110-5

[b23] Zhu Y. G. . Human mesenchymal stem cell microvesicles for treatment of *Escherichia coli* endotoxin-induced acute lung injury in mice. Stem cells 32, 116–125 (2014). 23939814 10.1002/stem.1504 PMC3947321

[b24] Gatti S. . Microvesicles derived from human adult mesenchymal stem cells protect against ischaemia-reperfusion-induced acute and chronic kidney injury. Nephrol Dial Transplant 26, 1474–1483 (2011). 21324974 10.1093/ndt/gfr015

[b25] Xin H. . Systemic administration of exosomes released from mesenchymal stromal cells promote functional recovery and neurovascular plasticity after stroke in rats. J Cereb Blood Flow Metab 33, 1711–1715 (2013). 23963371 10.1038/jcbfm.2013.152 PMC3824189

[b26] Silverman J. M. . An exosome-based secretion pathway is responsible for protein export from Leishmania and communication with macrophages. J Cell Sci 123, 842–852 (2010). 20159964 10.1242/jcs.056465

[b27] Masliah E., Terry R. D., Alford M. & DeTeresa R. Quantitative immunohistochemistry of synaptophysin in human neocortex: an alternative method to estimate density of presynaptic terminals in paraffin sections. J Histochem Cytochem 38, 837–844 (1990). 2110586 10.1177/38.6.2110586

[b28] Zhuang X. . Treatment of brain inflammatory diseases by delivering exosome encapsulated anti-inflammatory drugs from the nasal region to the brain. Mol Ther 19, 1769–1779 (2011). 21915101 10.1038/mt.2011.164 PMC3188748

[b29] Ryan J. M., Barry F. P., Murphy J. M. & Mahon B. P. Mesenchymal stem cells avoid allogeneic rejection. J Inflamm 2, 8 (2005). 10.1186/1476-9255-2-8 PMC1215510 16045800

[b30] Pols M. S. & Klumperman J. Trafficking and function of the tetraspanin CD63. Exp Cell Res 315, 1584–1592 (2009). 18930046 10.1016/j.yexcr.2008.09.020

[b31] Lancaster G. I. & Febbraio M. A. Exosome-dependent trafficking of HSP70: a novel secretory pathway for cellular stress proteins. J Biol Chem 280, 23349–23355 (2005). 15826944 10.1074/jbc.M502017200

[b32] Thery C., Amigorena S., Raposo G. & Clayton A. Isolation and characterization of exosomes from cell culture supernatants and biological fluids. Curr Protoc Cell Biol Chapter 3, Unit 3.22 (2006). 10.1002/0471143030.cb0322s30 18228490

[b33] An K. . Exosomes neutralize synaptic-plasticity-disrupting activity of Abeta assemblies *in vivo*. Mol Brain 6, 47 (2013). 24284042 10.1186/1756-6606-6-47 PMC4222117

[b34] Lai Y. L., Smith P. M., Lamm W. J. & Hildebrandt J. Sampling and analysis of cerebrospinal fluid for chronic studies in awake rats. J Appl Physiol Respir Environ Exerc Physiol 54, 1754–1757 (1983). 6409862 10.1152/jappl.1983.54.6.1754

[b35] Liu L. & Duff K. A technique for serial collection of cerebrospinal fluid from the cisterna magna in mouse. J Vis Exp 21, 960 (2008). 19066529 10.3791/960 PMC2762909

[b36] Mettlach G. . Enhancement of neuromuscular dynamics and strength behavior using extremely low magnitude mechanical signals in mice. J Biomech 47, 162–167 (2014). 24157062 10.1016/j.jbiomech.2013.09.024 PMC3881264

[b37] Zhao C. H. . Effects of dietary fish oil on learning function and apoptosis of hippocampal pyramidal neurons in streptozotocin-diabetic rats. Brain Res 1457, 33–43 (2012). 22542021 10.1016/j.brainres.2012.03.067

[b38] Won S. J. . Recurrent/moderate hypoglycemia induces hippocampal dendritic injury, microglial activation, and cognitive impairment in diabetic rats. J Neuroinflammation 9, 182 (2012). 22830525 10.1186/1742-2094-9-182 PMC3458941

[b39] Duarte J. M., Carvalho R. A., Cunha R. A. & Gruetter R. Caffeine consumption attenuates neurochemical modifications in the hippocampus of streptozotocin-induced diabetic rats. J Neurochem 111, 368–379 (2009). 19694901 10.1111/j.1471-4159.2009.06349.x

[b40] Grillo C. A. . Immunocytochemical analysis of synaptic proteins provides new insights into diabetes-mediated plasticity in the rat hippocampus. Neuroscience 136, 477–486 (2005). 16226381 10.1016/j.neuroscience.2005.08.019

[b41] Mezey E., Chandross K. J., Harta G., Maki R. A. & McKercher S. R. Turning blood into brain: cells bearing neuronal antigens generated *in vivo* from bone marrow. Science 290, 1779–1782 (2000). 11099419 10.1126/science.290.5497.1779

[b42] Tfilin M. . Mesenchymal stem cells increase hippocampal neurogenesis and counteract depressive-like behavior. Mol Psychiatry 15, 1164–1175 (2010). 19859069 10.1038/mp.2009.110

[b43] Scheff S. W., Price D. A., Schmitt F. A. & Mufson E. J. Hippocampal synaptic loss in early Alzheimer’s disease and mild cognitive impairment. Neurobiol Aging 27, 1372–1384 (2006). 16289476 10.1016/j.neurobiolaging.2005.09.012

[b44] Parkhurst C. N. & Gan W. B. Microglia dynamics and function in the CNS. Curr Opin Neurobiol 20, 595–600 (2010). 20705452 10.1016/j.conb.2010.07.002 PMC3708473

[b45] Nagayach A., Patro N. & Patro I. Astrocytic and microglial response in experimentally induced diabetic rat brain. Metab Brain Dis 29, 747–761 (2014). 24833555 10.1007/s11011-014-9562-z

[b46] Coleman E., Judd R., Hoe L., Dennis J. & Posner P. Effects of diabetes mellitus on astrocyte GFAP and glutamate transporters in the CNS. Glia 48, 166–178 (2004). 15378652 10.1002/glia.20068

[b47] Jing L. . Diabetes inhibits cerebral ischemia-induced astrocyte activation - an observation in the cingulate cortex. Int J Biol Sci 9, 980–988 (2013). 24163590 10.7150/ijbs.7251 PMC3807018

[b48] Amin S. N., Younan S. M., Youssef M. F., Rashed L. A. & Mohamady I. A histological and functional study on hippocampal formation of normal and diabetic rats. F1000Res 2, 151 (2013). 24555069 10.12688/f1000research.2-151.v1 PMC3901513

[b49] Duarte J. M., Agostinho P. M., Carvalho R. A. & Cunha R. A. Caffeine consumption prevents diabetes-induced memory impairment and synaptotoxicity in the hippocampus of NONcZNO10/LTJ mice. PLoS one 7, e21899 (2012). 22514596 10.1371/journal.pone.0021899 PMC3326010

[b50] Ahmadpour S. H. & Haghir H. Diabetes mellitus type 1 induces dark neuron formation in the dentate gyrus: a study by Gallyas’ method and transmission electron microscopy. Rom J Morphol Embryol 52, 575–579 (2011). 21655645

[b51] Sofroniew M. V. & Vinters H. V. Astrocytes: biology and pathology. Acta neuropathol 119, 7–35 (2010). 20012068 10.1007/s00401-009-0619-8 PMC2799634

[b52] Kimelberg H. K., Rutledge E., Goderie S. & Charniga C. Astrocytic swelling due to hypotonic or high K+ medium causes inhibition of glutamate and aspartate uptake and increases their release. J Cereb Blood Flow Metab 15, 409–416 (1995). 7713998 10.1038/jcbfm.1995.51

[b53] Suzuki M. & Raisman G. Multifocal pattern of postnatal development of the macroglial framework of the rat fimbria. Glia 12, 294–308 (1994). 7534272 10.1002/glia.440120406

[b54] Skutella T. & Nitsch R. New molecules for hippocampal development. Trends Neurosci 24, 107–113 (2001). 11164941 10.1016/s0166-2236(00)01717-3

[b55] Grapp M. . Choroid plexus transcytosis and exosome shuttling deliver folate into brain parenchyma. Nat Commun 4, 2123 (2013). 23828504 10.1038/ncomms3123

[b56] Xin H. . MiR-133b promotes neural plasticity and functional recovery after treatment of stroke with multipotent mesenchymal stromal cells in rats via transfer of exosome-enriched extracellular particles. Stem cells 31, 2737–2746 (2013). 23630198 10.1002/stem.1409 PMC3788061

[b57] Anders S. . Spatial properties of astrocyte gap junction coupling in the rat hippocampus. Philos Trans R Soc Lond B Biol Sci 369, 20130600 (2014). 25225094 10.1098/rstb.2013.0600 PMC4173286

[b58] Sofroniew M. V. Astrocyte barriers to neurotoxic inflammation. Nat Rev Neurosci 16, 249–263 (2015). 25891508 10.1038/nrn3898 PMC5253239

[b59] Diniz L. P. . Astrocyte-induced synaptogenesis is mediated by transforming growth factor beta signaling through modulation of D-serine levels in cerebral cortex neurons. J Biol Chem 287, 41432–41445 (2012). 23055518 10.1074/jbc.M112.380824 PMC3510841

[b60] Halassa M. M., Fellin T., Takano H., Dong J. H. & Haydon P. G. Synaptic islands defined by the territory of a single astrocyte. J Neurosci. 27, 6473–6477 (2007). 17567808 10.1523/JNEUROSCI.1419-07.2007 PMC6672436

