# Supplementary material for: Bone marrow-derived mesenchymal stem cells improve diabetes-induced cognitive impairment by exosome transfer into damaged neurons and astrocytes
Source: Sci Rep. 2016 Apr 22;6:24805. doi: 10.1038/srep24805 (PMC4840335; doi:10.1038/srep24805)
Supplement: Supplementary Information [file srep24805-s1.pdf]

Supplementary information

1. Supplementary Figure

2. Methods

3. References

Bone marrow-derived mesenchymal stem cells improve diabetes-induced cognitive impairment by exosome transfer into damaged neurons and astrocytes.

Masako Nakano, Kanna Nagaishi, Naoto Konari, Yuki Saito, Takako Chikenji, Yuka Mizue, Mineko Fujimiya

Supplementary Figure 1

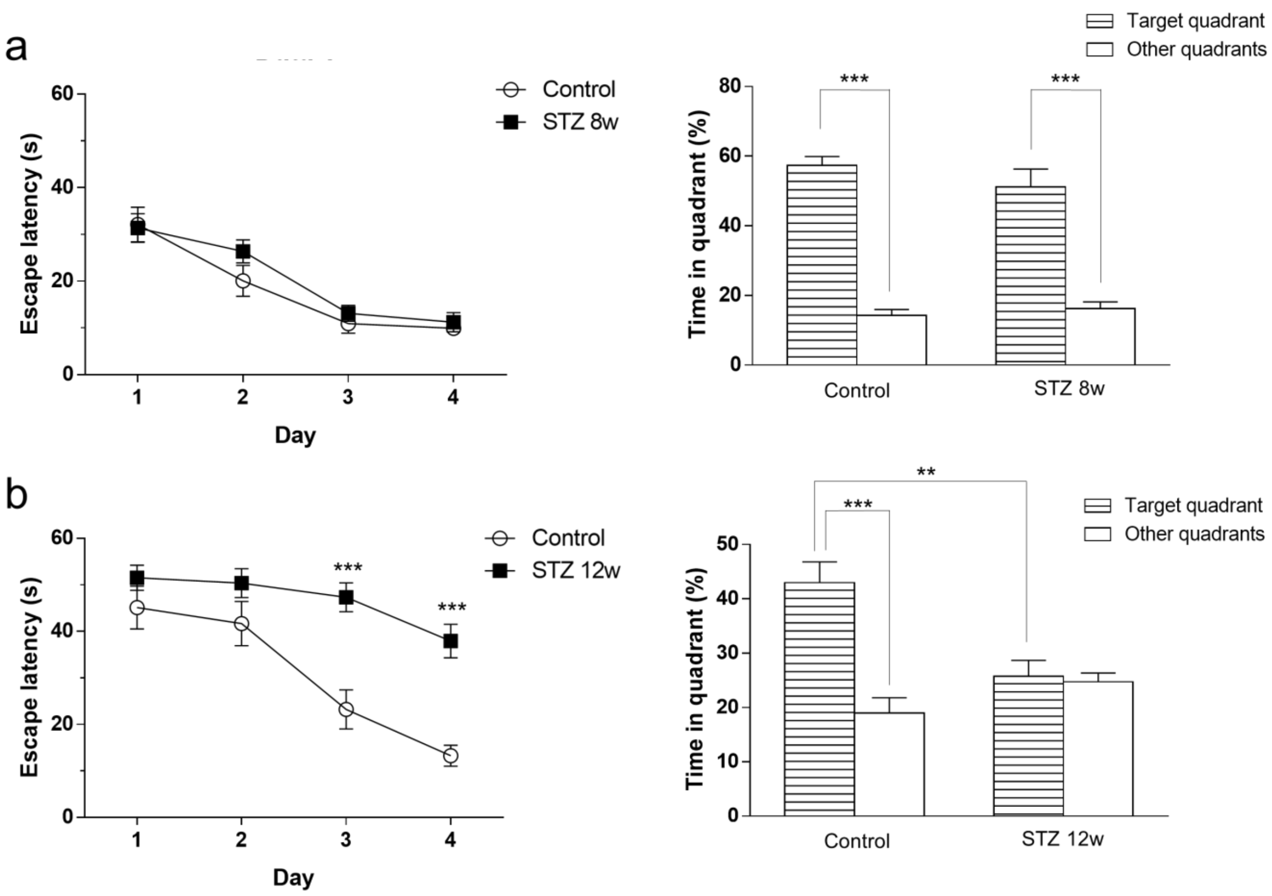

(**a**) MWM test conducted at 8 weeks after STZ injection. In the hidden training test, no difference is found in escape latency between Control and STZ 8w mice. Two-way ANOVA ($F(1,66) = 1.094$. P = 0.2994). Values are means ± s.e.m, n = 8-9. In the probe test, no difference is found in the target quadrant occupancy between Control and STZ 8w mice (P = 0.3127, unpaired two-tailed t-tests). Control and STZ 8w mice spend significantly more time in the target quadrant than any of the other quadrants (***P < 0.001, unpaired two-tailed t-tests). Values are means ± s.e.m. n = 8-9. (**b**) MWM test conducted at 12 weeks after STZ injection. In the hidden training test, STZ 12w mice exhibit longer escape latency on days 3 and 4 than control mice. ***P < 0.001, STZ 12w vs. control;

two-way ANOVA ($F(1,50) = 27.4$ $P < 0.0001$). Values are means ± s.e.m, n = 5-8. In the probe test, the target quadrant occupancy of STZ12w mice is significantly reduced compared with control mice (**$P < 0.01$, unpaired two-tailed t-tests). Control mice spend significantly more time in the target quadrant than any of the other quadrants (***$P < 0.001$, unpaired two-tailed t-tests). For STZ 12w mice, the time spent in the target quadrant is similar to that spent in the other quadrants ($P = 0.7511$, unpaired two-tailed t-tests). Values are means ± s.e.m. n = 5-8.

Supplementary Figure 2

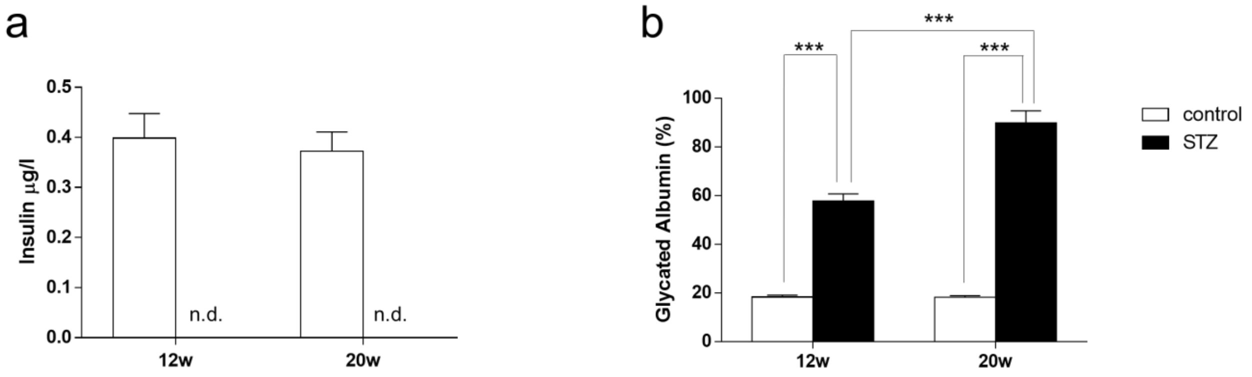

(**a**) Serum insulin levels at 12 and 20 weeks after STZ injection. Insulin is not detected in STZ mice. Values are means ± s.e.m, n = 6. (**b**) Glycated albumin levels at 12 and 20 weeks after STZ injection. Significant increase of glycated albumin level is found in STZ mice compared to controls (***$P < 0.001$, unpaired two-tailed *t*-tests). Glycated albumin levels at 20 weeks after STZ injection were significant increased compared to those of STZ 12 weeks mice (***$P < 0.001$, unpaired two-tailed *t*-tests).Values are means ± s.e.m, n = 6.

Supplementary Figure 3

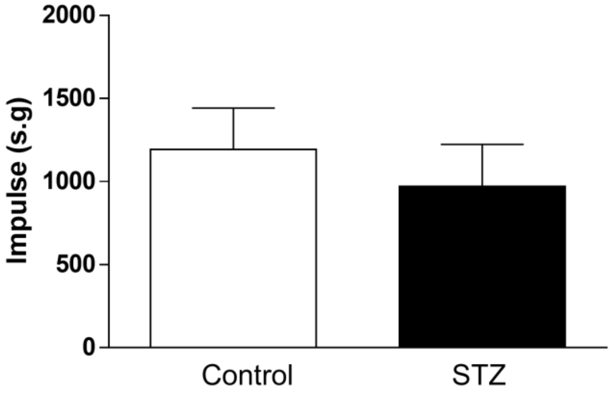

Hanging wire test. No difference is found between STZ and control mice (*P* = 0.556, unpaired two-tailed *t*-tests). Values are means ± s.e.m, n = 4-5.

Supplementary Figure 4

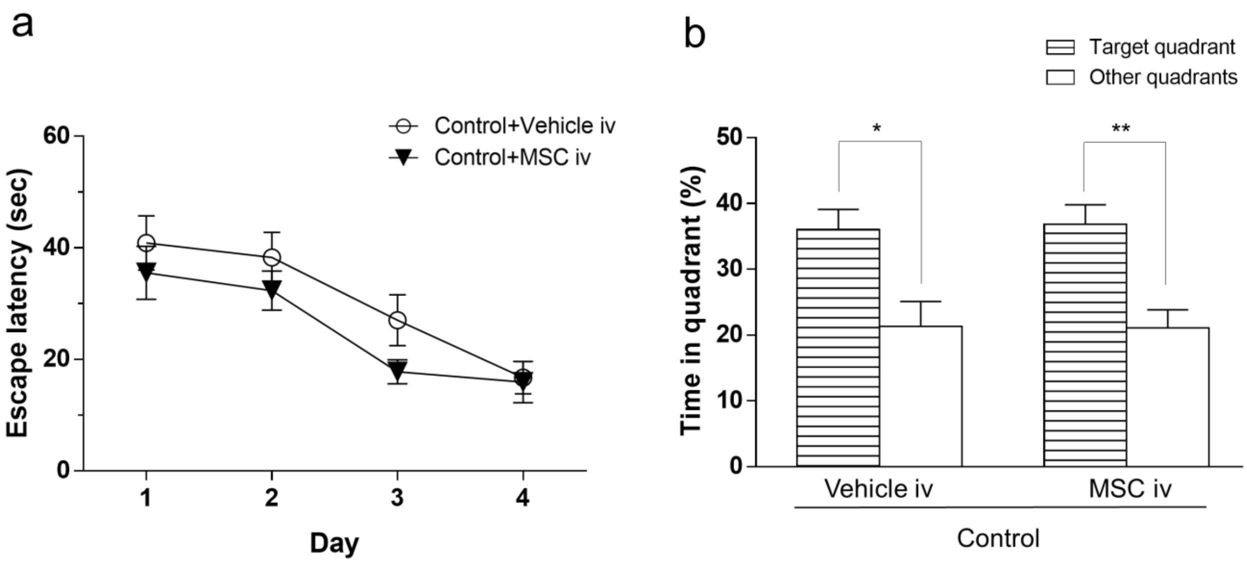

(**a**) MWM test. In the hidden training test, no difference is found in escape latency between control + vehicle and control + MSC mice. Two-way ANOVA ($F(1,42) = 3.2$. $P = 0.0809$). Values are means ± s.e.m, n = 5-6. (**b**) In the probe test, no difference is found in the target quadrant occupancy between control + vehicle and control + MSC mice ($P = 0.8541$, unpaired two-tailed *t*-tests). Control + vehicle and control + MSC mice spend significantly more time in the target quadrant than any of the other quadrants ($^*P < 0.05$, $^{**}P < 0.01$, unpaired two-tailed *t*-tests). Values are means ± s.e.m. n = 5-6.

Supplementary Figure 5

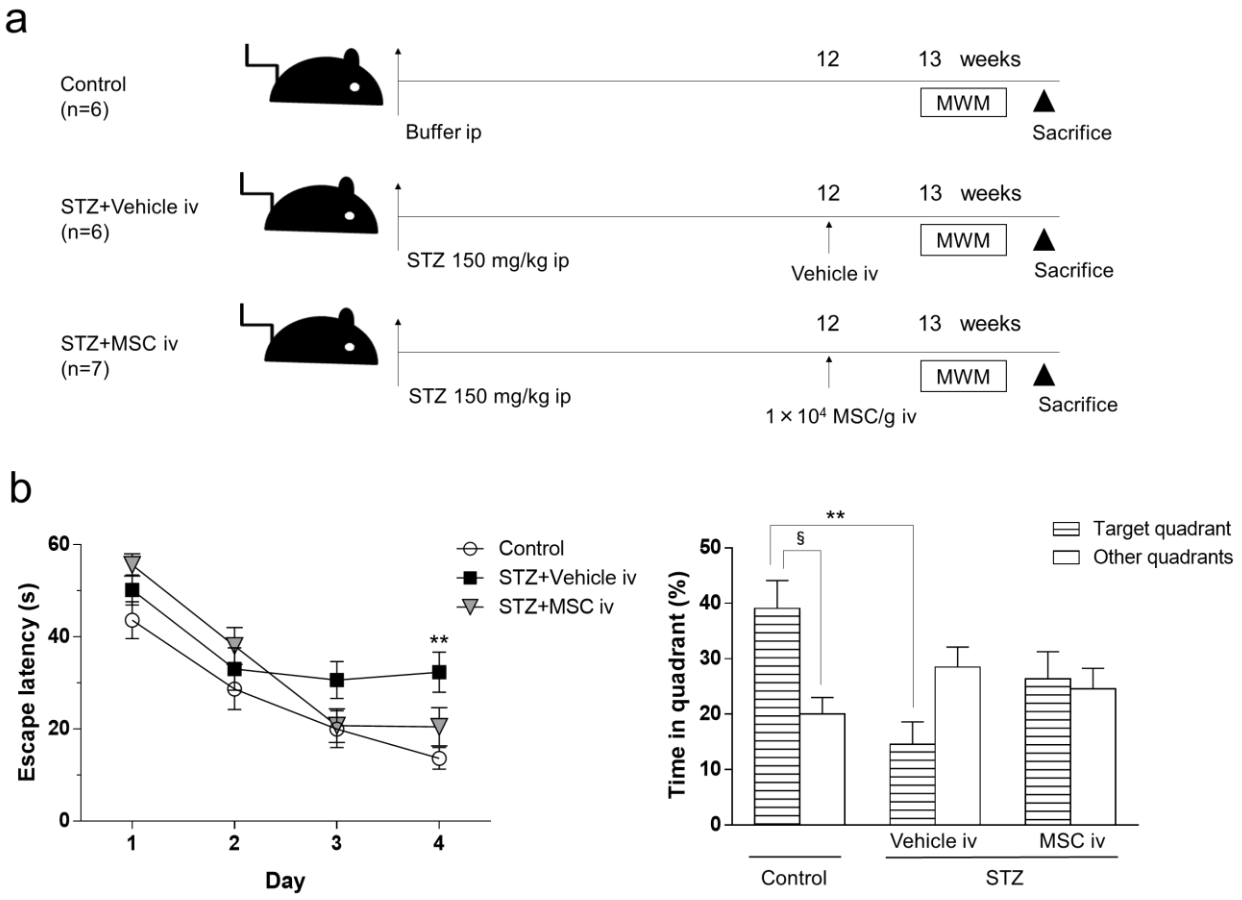

Intravenous injection of BM-MSCs into STZ-induced diabetic mice. (**a**) Experimental protocol. At 12 weeks after STZ injection, mice are injected iv with 1×10^4 MSCs/g body weight 1 times or with PBS for vehicle injection. 1 week after the injection, the MWM test is carried out. (**b**) MWM test. In the hidden training test, STZ + vehicle mice exhibit longer escape latency on days 4 than control mice. On the other hand, no difference is found in escape latency between STZ + vehicle and STZ + MSC mice. $^{**}P < 0.01$, STZ + vehicle vs. control, two-way ANOVA ($F(2,73) = 7.173$ $P = 0.0014$). Values are means ± s.e.m, n = 6-7. In the probe test, the target quadrant occupancy of STZ + vehicle mice is significantly reduced compared with control mice ($^{**}P < 0.01$, one-way ANOVA,

Bonferroni post-test). On the other hand, no difference is found in the target quadrant occupancy between STZ + vehicle and STZ + MSC mice. Control mice spend significantly more time in the target quadrant than any of the other quadrants (§ $P < 0.01$, unpaired two-tailed *t*-tests). For STZ + vehicle and STZ + MSC mice, the time spent in the target quadrant is similar to that spent in the other quadrants ($P > 0.05$, unpaired two-tailed *t*-tests). Swimming speed is similar among the 3 groups (control = 0.1503 ± 0.0068 m s^{-1}, STZ + vehicle = 0.1415 ± 0.0068 m s^{-1}, STZ + MSC = 0.1573 ± 0.0091 m s^{-1}; $P > 0.05$, one-way ANOVA). Values are means ± s.e.m. n = 6-7.

Supplementary Figure 6

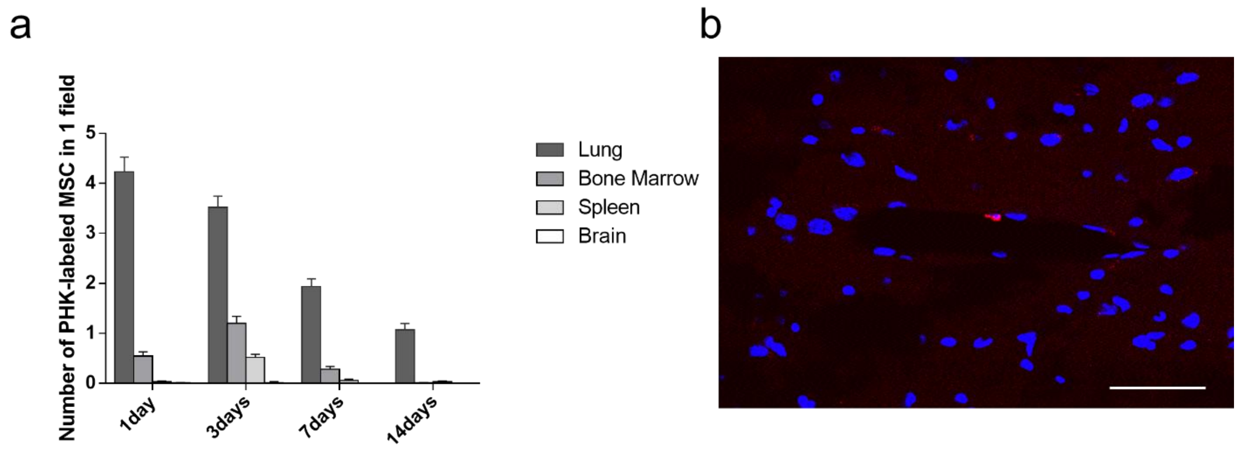

(**a**) Distribution of BM-MSCs in different organs. At 1 day after injection, PKH-labeled BM-MSCs are detected in the lung (4.23 ± 0.30 cells per 1 visual field), bone marrow (0.54 ± 0.09), spleen (0.03 ± 0.02), and brain (0.01± 0.01). At 3 days after injection, they are detected in the lung (3.52 ± 0.22), bone marrow (1.2 ± 0.14), spleen (0.52 ± 0.06), and brain (0.02 ± 0.01). At 7 days after injection, they are detected in the lung (1.93 ± 0.16), bone marrow (0.28 ± 0.06), and spleen (0.06 ± 0.02), but not in the brain. At 14 days after injection, they are detected in the lung (1.07 ± 0.13), bone marrow (0.01 ± 0.01), and spleen (0.03 ± 0.02), but not in the brain (0.00 ± 0.00). Values are means ± s.e.m. (**b**) Distribution of BM-MSCs in brain. Very few PKH-labeled BM-MSCs are observed at perivascular areas in the brain at 3 days after injection. Bar, 50 μm.

Supplementary Figure 7

a

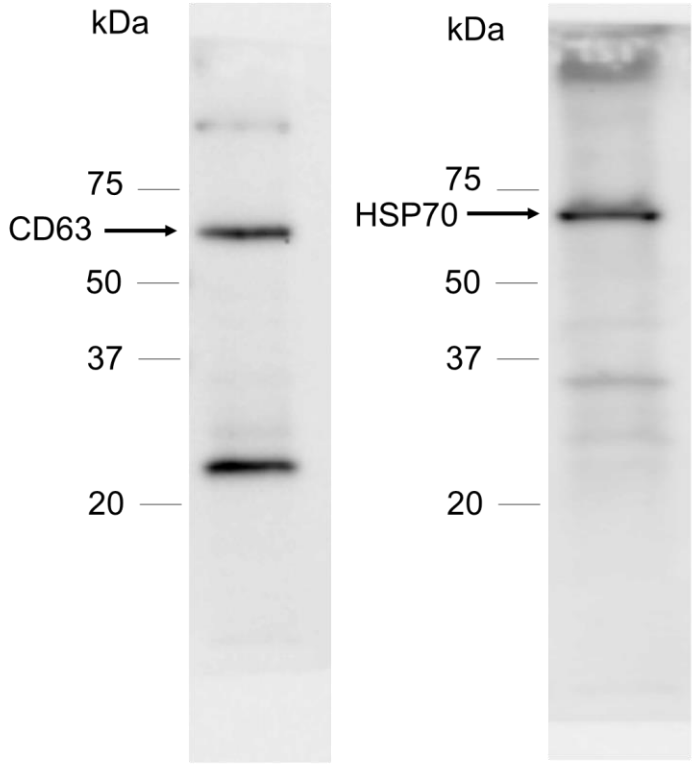

b

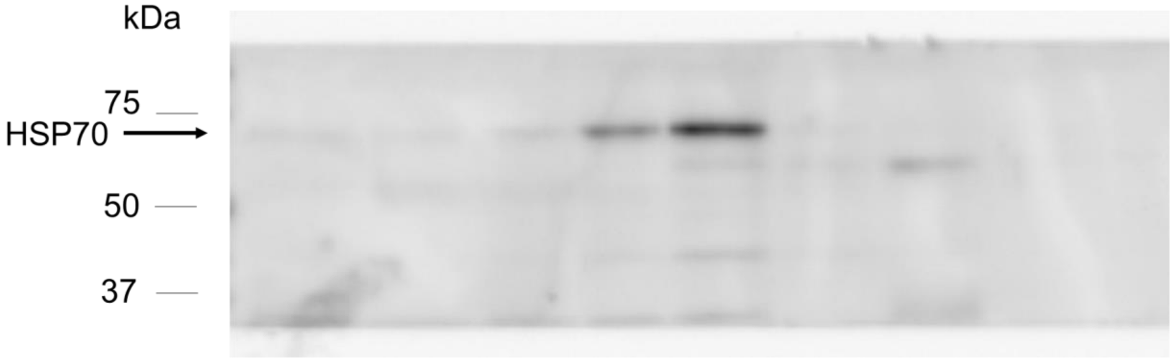

(**a**) The full-length blots in Figure 2a.

(**b**) The full-length blots in Figure 2b.

Supplementary Figure 8

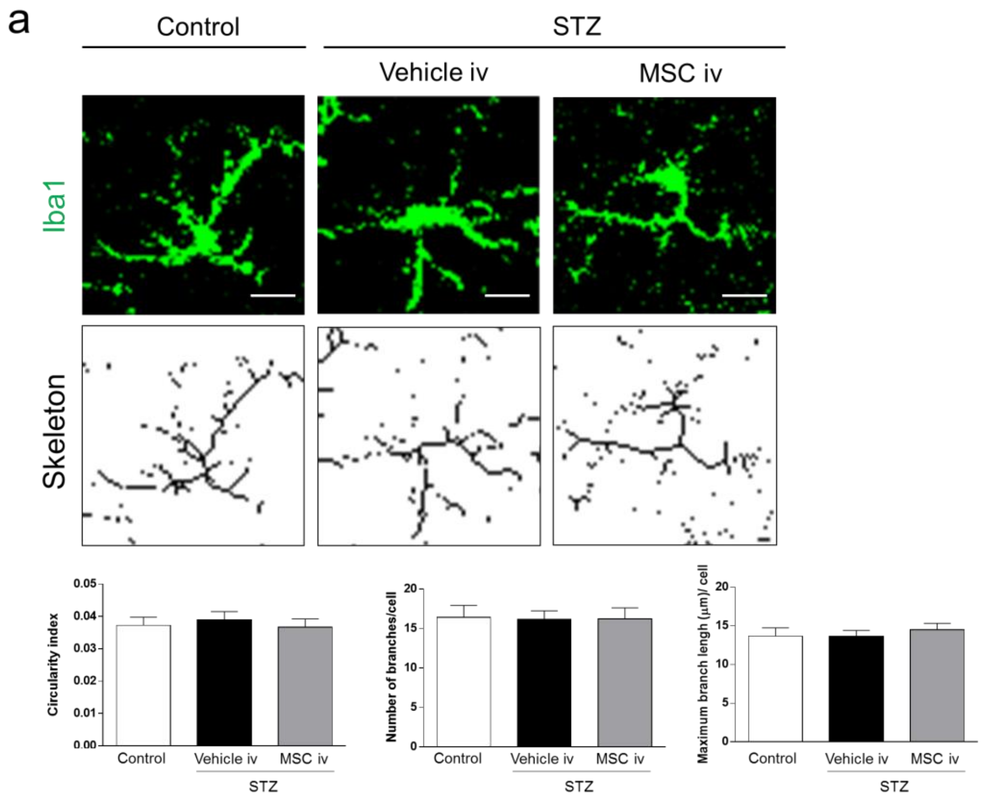

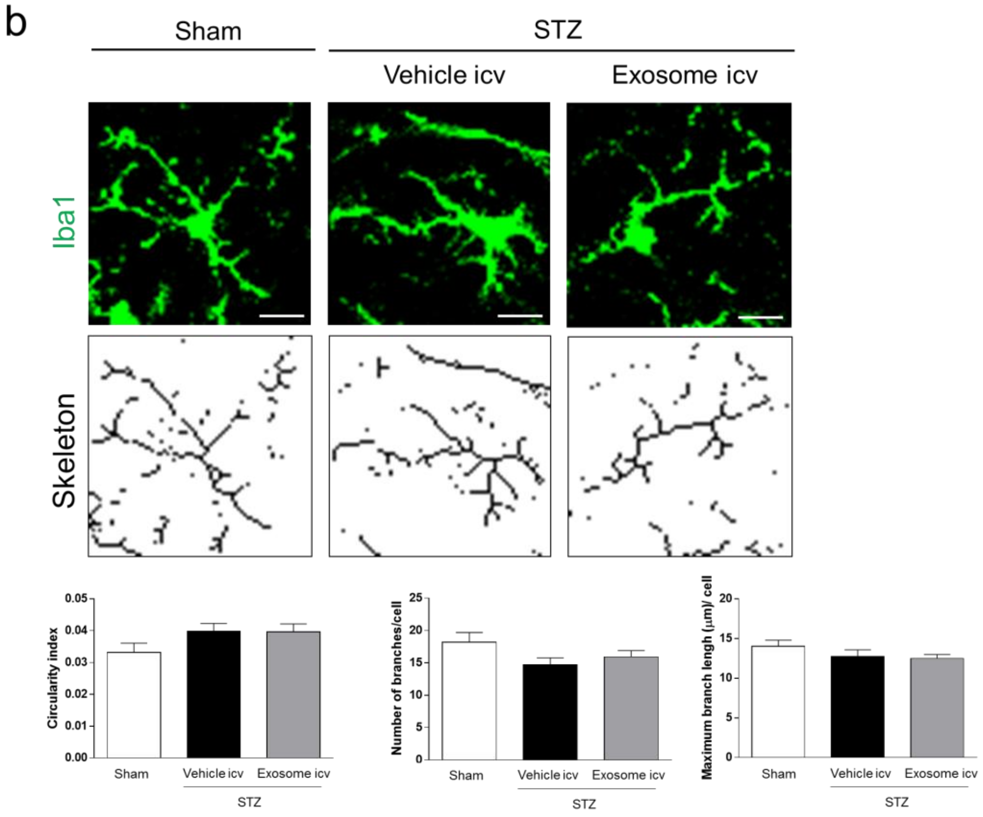

(**a**) Circularity, number of branches and maximum branch length of microglia among control, STZ + vehicle and STZ + MSC groups. No difference is found among three groups (*P* = 0.8071, 0.9901, 0.7427, respectively). One-way ANOVA. Values are means ± s.e.m, n = 4. Bar = 10 μm. (**b**) Circularity, number of branches and maximum branch length of microglia among control, STZ + vehicle, STZ + exosomes groups. No difference is found among three groups. (*P* = 0.1478, 0.1214, 0.2980, respectively). One-way ANOVA. Values are means ± s.e.m, n = 4. Bar = 10 μm.

Supplementary Figure 9

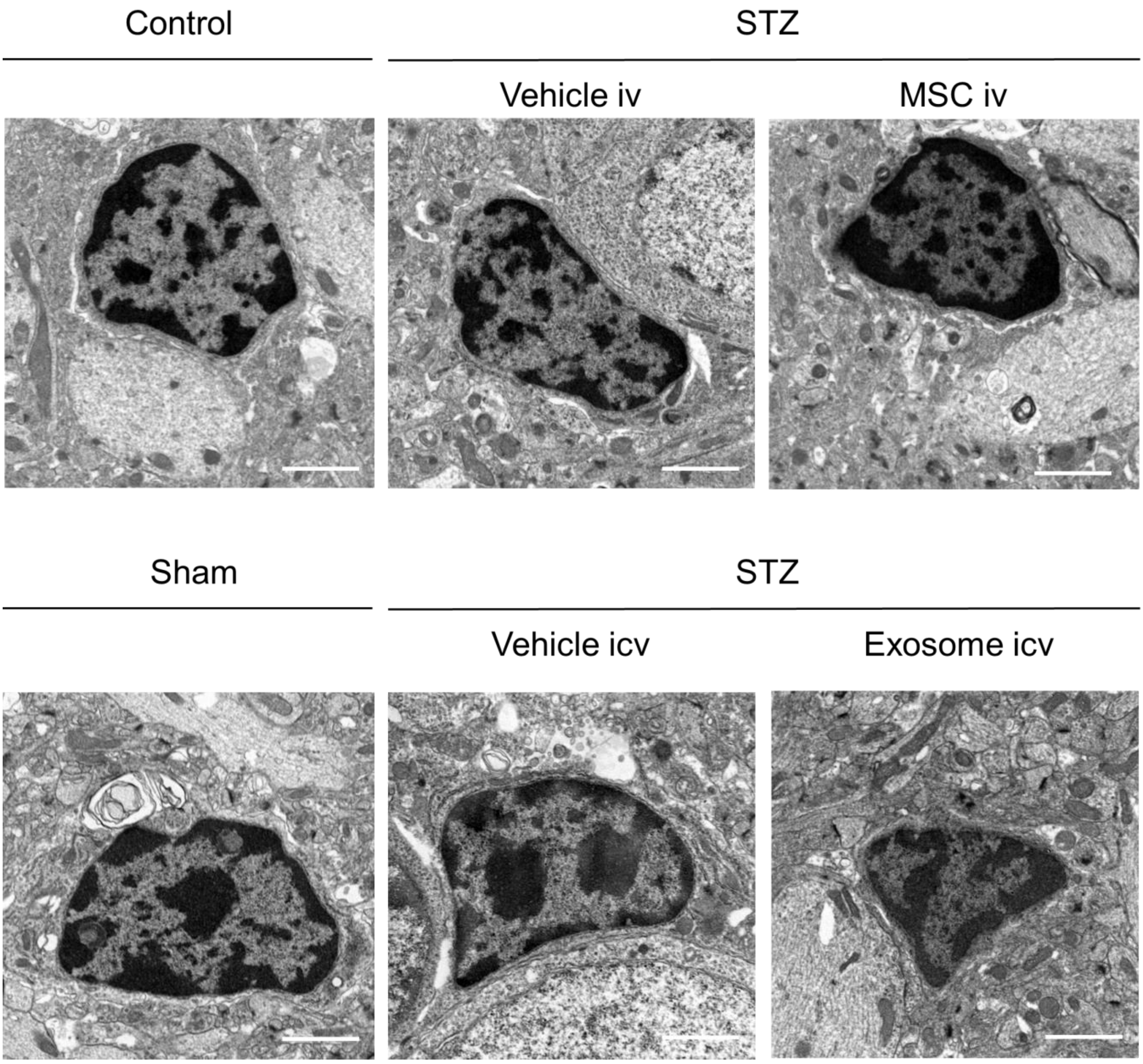

Ultrastructure of microglia. No difference is found in the morphology of microglia among control, STZ + vehicle and STZ + MSC, and also among sham, STZ + vehicle and STZ + exosomes groups. Bar = 2μm.

Supplementary Figure 10

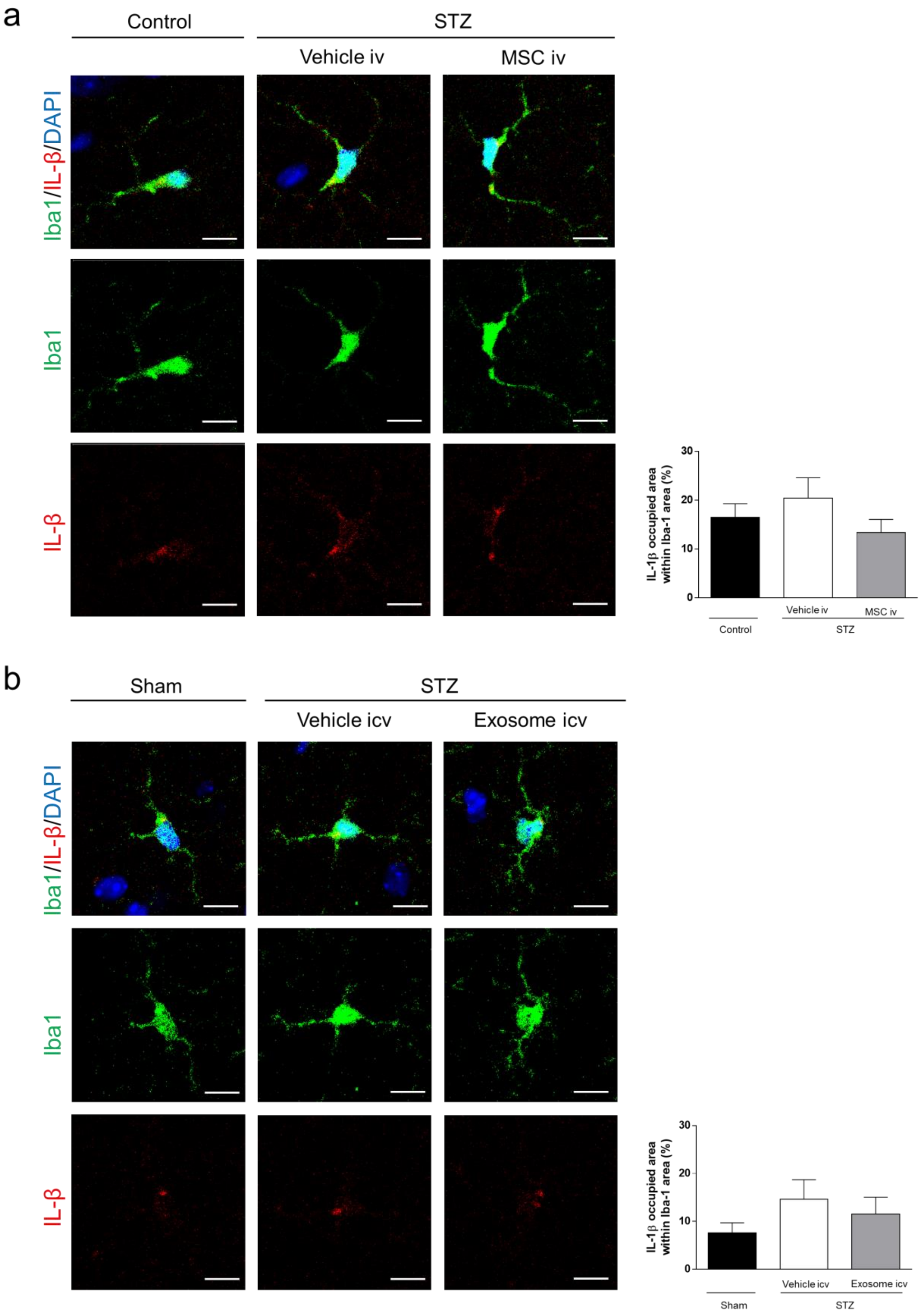

(**a**) IL-1β occupied area within Iba-1 among control, STZ + vehicle and STZ + MSC groups. No difference is found among three groups. (*P* = 0.3188, one-way ANOVA). Values are means ± s.e.m, n = 3-4. (**b**) Area occupied with IL-1β in Iba-1-positive area among sham, STZ + vehicle and STZ + exosomes groups. No difference is found among three groups. (*P* = 0.2964, one-way ANOVA). Values are means ± s.e.m, n = 4.

Supplementary Methods

Intravenous administration of fluorescence-labeled BM-MSCs.

BM-MSCs were stained with PKH26 (Sigma-Aldrich), according to the manufacturer's protocol. PKH26-labeled BM-MSCs (1×10^4 BM-MSCs/g body weight per animal suspended in 200 μl of PBS) were injected intravenously in STZ mice at 12 weeks after STZ injection. At 24 h, 3 days, 7 days, and 14 days after labeled BM-MSCs injections, lung, bone, spleen, and brain were fixed for immunohistochemistry. Frozen sections (20 μm) were stained with DAPI (Dojindo) and observed under confocal laser scanning microscopy. The number of PKH-positive cells within 100 randomly selected visual fields at 20 × magnification was counted in each organ per mouse, and the average was obtained.

Intracerebroventricular administration of fluorescence-labeled exosomes.

Exosomes obtained by multiple steps of ultracentrifugation were stained with PKH26 according to previous reports[1]. PKH-labeled exosomes (5 μg in 10 μL aCSF) were injected icv in STZ mice at 12 weeks after STZ injection. At 24 h after injection, mice were perfused and fixed, and then the right hemispheres (which was the side injected with exosomes) were cut into frozen sagittal sections (20 μm). Sections were incubated with primary antibodies (targeting NeuN, Neurofilament, GFAP, and Iba1), and then FITC-labeled anti-rabbit IgG (Millipore) and FITC-labeled anti-chicken

IgG (Abcam) were used as secondary antibodies. After staining nuclei by DAPI (Dojindo), the sections were observed under confocal laser scanning microscopy (Nikon A1). The number of PKH-positive exosomes merged with NeuN, Neurofilament, GFAP, and Iba1 was counted within 30 randomly selected visual fields in hippocampus fimbria at 40 × magnification, and the average was obtained.

Western blot analysis.

Proteins in the pellets collected with 100,000×*g* centrifugation and each pellet from the 9 fractions were denatured and separated on 12% SDS-polyacrylamide gels. After transfer onto a PVDF membrane, the membranes were treated with 5% skim milk. The primary antibodies used in this study were CD63 (diluted 1:1,000, System Bioscience, Mountain View, CA, USA) and HSP70 (diluted 1:1,000, System Bioscience); both are common exosome markers. For the secondary antibody, HRP-conjugated goat anti-rabbit IgG (diluted 1:20,000, System Bioscience) was used. The blots were visualized using a West Chemiluminescent Kit (Pierce, Rockford, IL, USA), and the images were detected by Las-3000 (Fujifilm Life Science, Kanagawa, Japan).

Exosome observation under electron microscopy.

Exosomes were observed by negative-stain electron microscopy. Briefly, a 10 μL suspension of isolated exosomes was fixed with 1% glutaraldehyde in PBS, applied to carbon-coated, 200 μm

mesh copper grids (Nisshin EM Corporation, Tokyo, Japan), and air dried for 1 h at room temperature. The grids were then stained with 2% uranyl acetate for 30 sec. Images were obtained by an electron microscope (H7650, HITACHI High-Technologies Corporation, Tokyo, Japan) at 80 kV.

Skeleton analysis

The microglial cell circularity and branch length were measured by skeleton analysis using the ImageJ (version 1.49, http://imagej.nih.gov/ij/) software, as described previously[2]. Briefly, confocal images were converted to binary images, then analyzed using the AnalyzeSkeleton plugin (http://fiji.sc/AnalyzeSkeleton) to obtain the circularity, the number of processes and maximum branch length for each cell.

Measurement of insulin and glycated albumin

Serum insulin levels were assayed using a mouse-specific insulin ELISA kit (Ultrasensitive Mouse Insulin ELISA; Mercodia, Uppsala, Sweden). Measurement of serum glycated albumin was delegated to Monolis, Inc. (Tokyo, Japan).

Hanging wire test

A metallic wire (55 cm length, 2 mm diameter) fixed between two vertical stands was maintained at a height of 35 cm. After the mouse was allowed to grasp the wire, the suspension time

until the animal fell down was measured. The test was performed in 3 trials per mouse, and each trial had a time limit of 180 sec. In experiments, the maximum hanging time was used as Hanging time (sec), and the Holding Impulse [(s*g) = Body mass (grams) x Hanging Time (sec)] was calculated to diminish the effect of body weight.

References

1 Yuyama, K. *et al.* Decreased amyloid-beta pathologies by intracerebral loading of glycosphingolipid-enriched exosomes in Alzheimer model mice. *J Biol Chem* **289**, 24488-24498 (2014).

2 Madeira, M. H. *et al.* Adenosine A2AR blockade prevents neuroinflammation-induced death of retinal ganglion cells caused by elevated pressure. *J Neuroinflammation* **12**, 115 (2015).
